# Synthesis of β-triazolylenones via metal-free desulfonylative alkylation of *N*-tosyl-1,2,3-triazoles

**DOI:** 10.3762/bjoc.17.66

**Published:** 2021-03-31

**Authors:** Soumyaranjan Pati, Renata G Almeida, Eufrânio N da Silva Júnior, Irishi N N Namboothiri

**Affiliations:** 1 Department of Chemistry, Indian Institute of Technology Bombay, Mumbai, 400 076, India; 2 Institute of Exact Sciences, Department of Chemistry, Federal University of Minas Gerais, CEP 31270-901, Belo Horizonte, MG, Brazil

**Keywords:** azoles, cycloaddition, enones, heterocycles, 1,2,3-triazoles

## Abstract

Desulfonylative alkylation of *N*-tosyl-1,2,3-triazoles under metal-free conditions leading to β-triazolylenones is reported here. The present study encompasses the synthesis of triazoles with a new substitution pattern in a single step from cyclic 1,3-dicarbonyl compounds and *N*-tosyl triazole in moderate to high yields. Our synthesis takes place with complete regioselectivity as confirmed by crystallographic analysis which is rationalized by a suitable mechanistic proposal. This method provides an efficient, versatile and straightforward strategy towards the synthesis of new functionalized 1,2,3-triazoles.

## Introduction

**Scheme 1:**
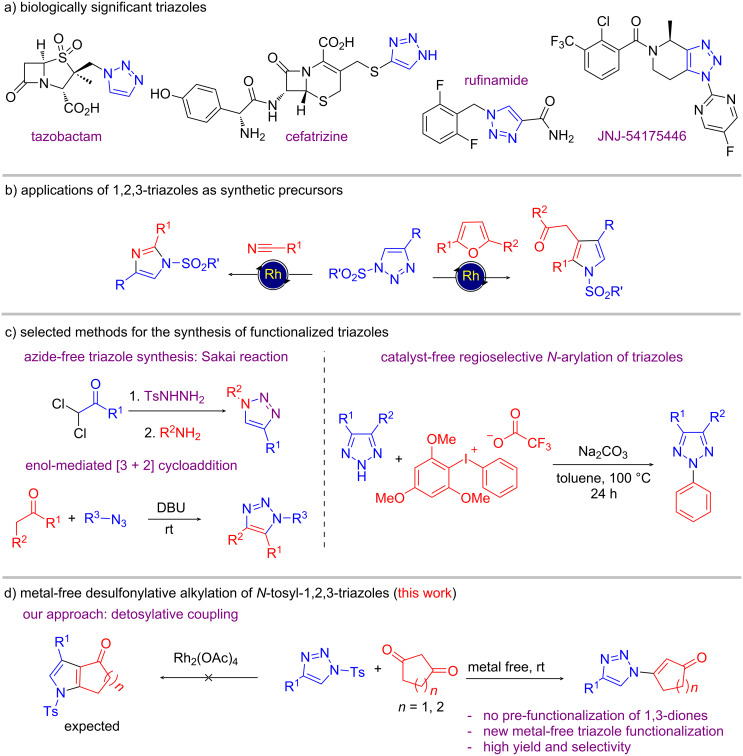
Synthesis, functionalization and applications of triazoles.

1,2,3-Triazoles are significant non-natural heterocyclic scaffolds with extensive applications in biochemistry, agrochemistry and materials chemistry [1–5]. This class of heterocycles presents important biological properties, such as antiviral, anti-inflammatory, antimicrobial etc. and are considered as key building blocks in pharmaceutical industry [6–9]. Thus, they marked their presence as prominent scaffolds in many drug molecules such as tazobactam, cefatrizine, rufinamide and JNJ-54175446 (Scheme 1a) [10].

In addition to their biological activities, triazolic compounds are widely employed in organic synthesis and have outstanding synthetic versatility. In this sense, extensive scientific research has been conducted using triazoles as synthetic precursors in denitrogenative transannulation reactions under metal-catalysed conditions to form other heterocycles such as functionalized pyrroles, imidazoles and pyridines (Scheme 1b) [11–13].

The traditional method for the synthesis of triazole unit is the Huisgen 1,3-dipolar cycloaddition between azides and alkynes [14–15]. However, the formation of the nitrogenated azoles by the classical Huisgen methodology is slow due to its high activation energies and also lack of regiochemical control, in general, leading to a mixture of 1,4- and 1,5-regioisomers of 1,2,3-triazoles. Later, Sharpless and Meldal have independently developed a copper-catalysed azide–alkyne cycloaddition that accelerated the rate of the reaction and allowed the selective preparation of 1,5-disubstituted 1,2,3-triazoles [16–19].

As noted above, a wide range of methods are available in the literature for the efficient synthesis of triazoles with different substitution pattern. One important methodology developed by the Sakai group involved the reaction of α,α-dichloroketone, tosyl hydrazide and primary amine [20]. However, in this case, the unstable α,α-dichlorohydrazone intermediate had to be isolated which paved the way for further modification of the protocol (Scheme 1c).

Direct functionalization of triazoles is an alternative strategy to access triazoles with the desired substitution pattern. However, such approaches are complicated by a low energy barrier between the *N*^1^ and *N*^2^ tautomers in solution leading to poor *N*^1^/*N*^2^ selectivity, for instance, in the direct *N*-alkylation of triazoles [21–23]. Despite these challenges, a number of *N*^1^- and *N*^2^-selective alkylation methods have been developed employing transition metal catalysts which include Au-catalysed desulfonylative coupling *N*-tosyl-1,2,3-triazoles with alkynes and Rh catalysed *N*^1^ and *N*^2^ selective alkylations [24–25].

As for metal-free approaches, besides synthesizing *N*-alkylated triazoles via 1,3-dipolar cycloaddition of alkyl azide with enols generated from carbonyl compounds under transition metal-free conditions [26], a direct functionalization of triazoles under metal-free conditions has been reported. These include the Broensted acid-catalysed *N*^2^ alkylation [27], organocatalytic *N*^1^ alkylation [28–29], *N*^2^-arylation using hypervalent iodine (Scheme 1c) [30], *N*^2^-alkylation involving radical intermediate [31], pyridine-*N*-oxide-mediated *N*^1^-arylation [32], NIS-mediated *N*^2^-arylation [33], etc. Although these are significant advances towards metal-free functionalization of triazoles, many of them suffer from poor regioselectivity. Therefore, a new method for *N*^1^-selective alkylation of the triazole moiety under simple, mild and metal-free conditions is highly desirable.

From another perspective, compounds containing a 1,3-dicarbonyl moiety are essential building blocks in organic synthesis whose reactivity is well-established in the literature [34–35]. Besides, these are the precursors of β-enamines which are employed for the synthesis of many bioactive heterocycles [36]. These are also important precursors of diazo adducts which are used in insertion, cyclopropanation, and various rearrangements to construct various cyclic as well as acyclic moieties under metal-catalysed conditions [37–38]. On the contrary, under basic conditions, these diazo compounds undergo [3 + 2] cycloadditions with suitable substrates to render various nitrogen-rich heterocycles [39]. In addition to their synthetic importance, these are frequently encountered as ligands in many metal complexes [40–43]. Our group have also employed 1,3-dicarbonyl compounds as binucleophiles for the construction of various carbocycles, heterocycles as well as in asymmetric catalysis [44–50]. Our initial objective to trap the aza vinyl rhodium carbenoid using 1,3-dicarbonyl compounds to form pyrazolone was unsuccessful which instead led to the formation of an unexpected product, i.e., β-triazolylenone. Being inspired by the results, we intended to use 1,3-dicarbonyl compounds as detosylative alkylating agents that would lead to the formation of β-triazolylenones in a highly regioselective manner under mild conditions (Scheme 1d).

## Results and Discussion

In order to execute our idea, triazole **1a** and 1,3-cyclohexanedione (**2a**) were selected as our model substrates and the reaction was performed using 4 mol % of Rh_2_(OAc)_4_ in chloroform under reflux conditions which afforded the β-1,2,3-triazolylcyclohexenone **3a** in 66% yield (Table 1, entry 1). The alternative approach for the synthesis of such triazole moiety **3a** is the [3 + 2] cycloaddition between alkynes and the corresponding azides. However, the major disadvantage of such a strategy is the use of 3-azidoenone which is difficult to handle owing to its explosive nature. Therefore, our method provides an easy pathway to synthesize such triazolylenones.

**Table 1:** Optimization studies.^a^

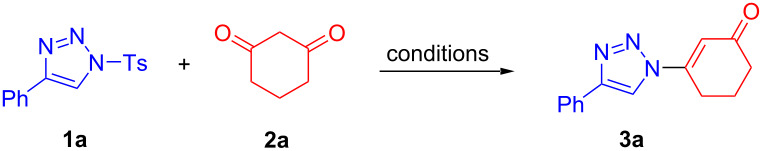					

Entry	Catalyst (mol %)	Solvent	Temp. (°C)	Time (h)	Yield (%)^b^

1	Rh_2_(OAc)_4_ (4)	CHCl_3_	60	2.5	66
2	Cu(OAc)_2_ (4)	CHCl_3_	60	6	65
3	…	CHCl_3_	60	5	49
**4**	**…**	**CHCl_3_**	**rt**	**24**	**78**
5	…	THF	rt	48	NR
6	…	EtOAc	rt	48	trace
7	…	toluene	rt	72	trace
8	…	CH_2_Cl_2_	rt	96	56
9	…	1,2-DCE	rt	72	trace
10	…	2-MeTHF	rt	72	^c^
11	…	isopropanol	rt	72	^c^
12	…	acetone	rt	72	^c^

^a^Performed by using 0.1 mmol 4-phenyl-*N*-tosyl-1*H*-1,2,3- triazole (**1a**) and 0.1 mmol of cycloalkan-1,3-dione **2**. ^b^After silica gel column chromatography. ^c^**1a** decomposed.

Inspired by this result, we proceeded to optimize the reaction conditions to further improve the yield. Replacement of rhodium by copper (II) acetate slowed down the reaction with a marginal change in the yield of the product **3a** (Table 1, entry 2). Surprisingly, when the reaction was conducted in the absence of any metal catalyst, the reaction proceeded sluggishly and the coupling product **3a** was isolated in 49% yield (Table 1, entry 3). This suggested that the reaction could also proceed even in the absence of metal catalyst. However, the lower yield of the product **3a** might be attributed to certain side products obtained at high temperature as evident from TLC analysis. To avoid such side reactions, the reaction was performed at room temperature and to our delight, the product **3a** was obtained in 78% yield (Table 1, entry 4). For further improvement in the yield, the reaction was carried out in different solvents such as THF, EtOAc, toluene, DCM, 1,2-DCE, 2-MeTHF, isopropanol and acetone. Unfortunately, these attempts led to inferior results (Table 1, entries 5–12). Since, there was no further improvement in the yield, the conditions described in Table 1, entry 4 were considered as the best to generalize the scope of the reaction.

**Scheme 2:**
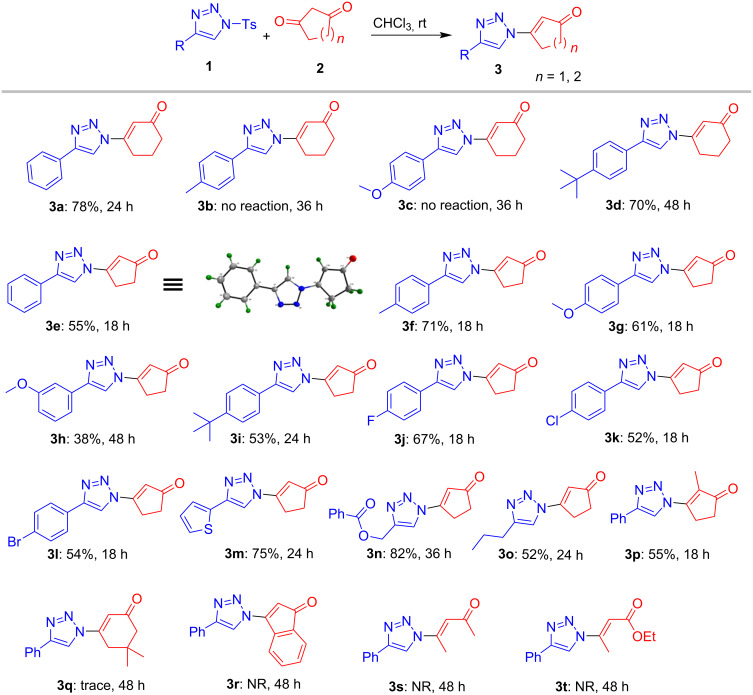
The reaction was performed using 0.2 mmol *N*-tosyl-1,2,3-triazole **1** and 0.2 mmol of cyclohexyl-1,3-dione **2**. Yields are determined after silica gel column chromatography. NR = no reaction.

At the outset, various triazoles **1** were screened under the optimized conditions (Scheme 2). As mentioned earlier, the model triazole **1a** afforded the product **3a** in 78% yield within 24 h. But to our surprise, triazoles **1b** and **1c** containing electron donating 4-tolyl and 4-methoxyphenyl groups did not deliver the products **3b** and **3c**, respectively, even after prolonged reaction time. This is attributable to the greater reactivity of their corresponding triazolyl anion which preferred protonation over Michael addition (see mechanism, Scheme 4, vide infra). On the other hand, 4-*tert*-butyl analog **1d** underwent the reaction smoothly to form the corresponding product **3d** in 70% yield. However, due to the inconsistent results with cyclohexanedione **2a**, further scope was investigated by employing cyclopentane-1,3-dione **2b**. The model triazole **1a** furnished the product **3e** in 55% yield within 18 h. To our delight, triazoles **1b** and **1c**, bearing 4-tolyl and 4-methoxyphenyl groups, which did not react with 1,3-cyclohexanedione **2a** reacted smoothly with cyclopentane-1,3-dione **2b** to deliver the products **3f** and **3g** in 71% and 61% yields, respectively. The reaction of mild electron withdrawing 3-methoxyphenyl-1,2,3-triazole **1e** led to product **3h** in low yield (38%) whereas the corresponding 4-*tert*-butylphenyl-1,2,3-triazole **1f** afforded the product **3i** in 53% yield. Later, the reaction was performed using various haloaryltriazoles, such as 4-fluorophenyl **1f**, 4-chlorophenyl **1g** and 4-bromophenyl **1h**, which also gave the corresponding products **3j**, **3k** and **3l** in 67%, 52% and 54%, respectively.

Further, the heteroaryl derivative thienyltriazole **1i** also reacted well to afford the product **3m** in 75% yield. While the benzoyloxymethyltriazole **1j** furnished the product **3n** in excellent (82%) yield, the performance of another alkyltriazole **1k** was less impressive giving the corresponding product **3o** only in moderate (52%) yield. The reaction of model triazole **1a** with 2-methyl-1,3-cyclopentanedione (**2c**) also led to the product **3p** in moderate (55%) yield. Unfortunately, the reaction was not successful with dimedone (**2d**), 1,3-indanedione (**2e**) and acyclic 1,3-dicarbonyl compounds such as acetylacetone (**2e**) and ethyl acetoacetate (**2f**).

The structure and regiochemistry of all the products were confirmed by detailed analysis of their spectral data (IR, ^1^H, ^13^C and Mass) which were further unambiguously established by single crystal X-ray analysis of a representative compound **3e** (Scheme 2 and Supporting Information File 1).

**Scheme 3:**
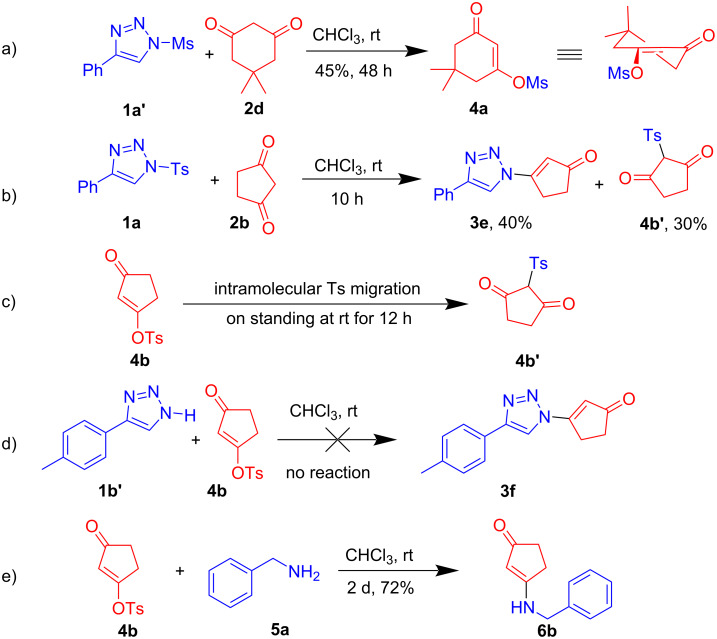
Control experiments.

Although triazole **1a** reacted with cyclohexanedione **2a** (vide supra), its reaction with dimedone **2d** provided a complex mixture. Therefore, we employed triazole **1a’** bearing a mesyl group for the reaction with **2d** (Scheme 3a). Surprisingly, this reaction delivered the 5,5-dimethyl-3-oxocyclohex-1-en-1-yl methanesulfonate (**4a**), instead of the expected β-triazolylenone **3q**, in 45% yield. In order to further ascertain the reaction mechanism, the crude reaction mixture of **1a** with **2b** after 10 h was analysed by NMR which suggested the formation of C-tosyl intermediate **4b'** besides the expected product **3e** (Scheme 3b). The two compounds were later purified and characterized. Subsequently, the cyclopentan-1,3-dione derived *O*-tosyl intermediate **4b** was prepared following a literature procedure [51]. Even though the *O*-tosyl intermediate **4b** was stable at low temperature (0 °C), surprisingly, it got converted to C-tosyl intermediate on standing overnight at room temperature (Scheme 3c). However, treatment of this *O*-tosyl intermediate **4b** with triazole **1b'** did not afford the expected product **3f** (Scheme 3d). This suggests that triazole **1b'** is not the active nucleophile in this transformation. However, when the *O*-tosyl intermediate **4b** was treated with a more nucleophilic amine, namely benzyl amine **5a**, it indeed afforded the Michael addition–elimination product **6b** in good yield. These results provided crucial evidence for the mechanism of the reaction which suggested that β-sulfonyloxyenone could be the key intermediate in the formation of β-triazolylenone **3**.

**Scheme 4:**
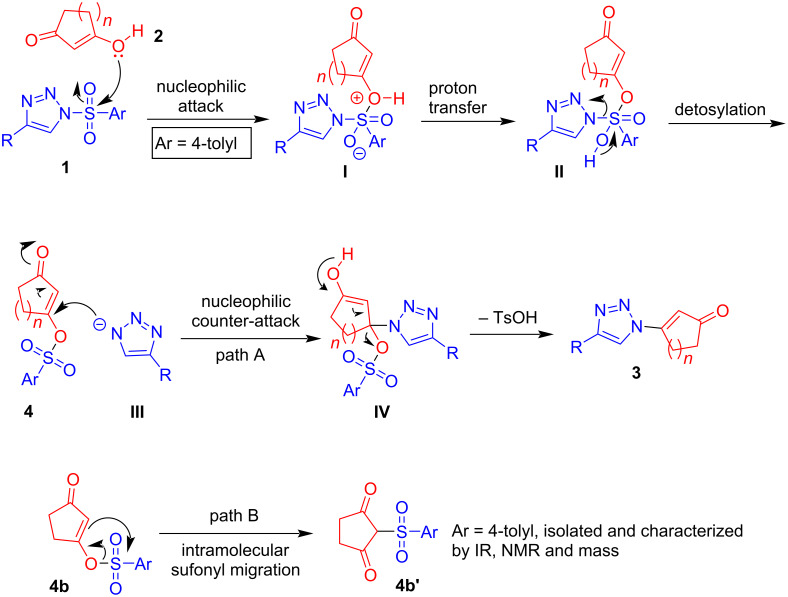
Mechanistic proposal for the formation of β-triazolylenones.

Based on the above control experiments, the following mechanism is proposed. Initially, the enol form of 1,3-dicarbonyl **2** attacks the sulfonyl group in **1** to form intermediate **I** which later undergoes proton transfer to form intermediate **II**. Cleavage of the N–S bond in intermediate **II** generates the β-*O*-tosylcycloalkenone **4** and triazolyl anion **III**. Subsequent counter-attack of the triazolyl anion **III** on the enone intermediate **4** (path A), followed by elimination of OTs affords the corresponding β-triazolylenone **3** (Scheme 4) [52]. The β-*O*-tosylcyclopentenone intermediate **4b** can also undergo intramolecular tosyl migration to form a stable C-tosylated product **4b’** (see also Scheme 3b and c). This might also be attributed to the lower yields obtained in certain cases.

**Figure 1:**
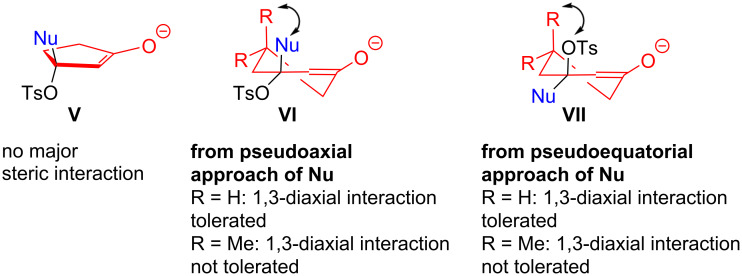
Nucleophilic addition to 5- and 6-membered cyclic tosyloxyenones.

It may be noted that the outcome of the reaction is highly dependent on the nature of the 1,3-dicarbonyl compound. In the case of cyclic-1,3-dicarbonyls, cyclopentane-1,3-dione **2b** reacts smoothly in almost all cases whereas the scope of cyclohexane-1,3-dione **2a** is limited. On the contrary, dimedone (**2d**) did not react with *N*-tosyl-1,2,3-triazole, but reacted with *N*-mesyl-1,2,3-triazole to form the 5,5-dimethyl-3-oxo-cyclohex-1-en-1-yl methanesulfonate intermediate **4a**. The highly substrate-dependent nature of the reaction can be explained by taking both hydrogen bonding and steric factors into consideration. In the solid as well as in the solution state, all the three above-mentioned 1,3-dicarbonyls exist in the enol form which are considerably stable and therefore undergo tosylation easily to form the corresponding *O*-tosylenone intermediates. The five membered tosyloxyenone intermediate is a planar molecule and therefore free of any major steric crowding (Figure 1). Hence, the incoming nucleophile can easily attack the β-position without any difficulty. On the other hand, the six membered *o*-tosyl intermediate is likely to exist in twist-boat conformation. When R = H, the pseudoaxial approach of the nucleophile towards the β-position leading to the enolate intermediate **VI** (R = H) bearing pseudoequatorially oriented OTs group or the pseudoequatorial approach of the nucleophile leading to the intermediate **VII** (R = H) bearing pseudoaxially oriented OTs group suffers from only limited steric crowding (1,3-diaxial interaction). In the case of dimedone **2d**, the two bulky methyl groups impart greater steric hindrance (1,3-diaxial interaction) to the incoming nucleophile in the event of pseudoaxial approach leading to intermediate **VI** (R = Me) and to the axial OTs group in the resulting intermediate **VII** (R = Me) in the event of equatorial approach of the nucleophile. Therefore, the reaction proceeds in the case of 1,3-cyclohexanedione (**2a**), but stops at the tosyl/mesyl migration step and does not proceed further in the case of dimedone (**2d**). Unlike cyclic 1,3-dicarbonyls, the acyclic 1,3-dicarbonyls possess intramolecular hydrogen bonding and are in rapid equilibrium with their keto-form. In polar solvents, the stability of the enol form is further decreased and, therefore, the keto-enol equilibrium lies more towards the keto-form [53–56]. Presumably for this reason, the acyclic 1,3-dicarbonyls did not react in the desired way under our experimental conditions.

## Conclusion

In conclusion, we have developed a metal-free and catalyst-free approach for the desulfonylative coupling of *N*-tosyl-1,2,3-triazoles with cyclic 1,3-diketones to form various β-triazolylenones. Although the scope of the 1,3-dicarbonyl compound is limited, the protocol is very convenient and useful as it employs very mild reaction conditions. It is also highly regioselective and `affords only *N*^1^-alkylated products in moderate to excellent yields.

## Supporting Information

File 1 Experimental details and characterization data of new compounds.

File 2 Copies of NMR spectra.

File 3 Crystallographic data for compound **3e**.
