# Supplementary material for: Synthesis of β-triazolylenones via metal-free desulfonylative alkylation of N-tosyl-1,2,3-triazoles
Source: Beilstein J Org Chem. 2021 Mar 31;17:762–70. doi: 10.3762/bjoc.17.66 (PMC8022205; doi:10.3762/bjoc.17.66)
Supplement: File 1 — Experimental details and characterization data of new compounds. [file Beilstein_J_Org_Chem-17-762-s001.pdf]

BEILSTEIN JOURNAL OF ORGANIC CHEMISTRY

Supporting Information

for

Synthesis of β-triazolylenones via metal-free desulfonylative alkylation of *N*-tosyl-1,2,3-triazoles

Soumyaranjan Pati, Renata G. Almeida, Eufrânio N. da Silva Júnior
and Irishi N. N. Namboothiri

Beilstein J. Org. Chem. **2021,** *17,* 762–770. doi:10.3762/bjoc.17.66

Experimental details and characterization data of new compounds

License and Terms: This is a supporting information file under the terms of the Creative Commons Attribution License (https://creativecommons.org/licenses/by/4.0). Please note that the reuse, redistribution and reproduction in particular requires that the author(s) and source are credited and that individual graphics may be subject to special legal provisions.
The license is subject to the *Beilstein Journal of Organic Chemistry* terms and conditions: (https://www.beilstein-journals.org/bjoc/terms)

Entry		Page
1	General experimental details	S1
2	General procedure	S1
3	Experimental data	S1
4	References	S6

General experimental details. A Buchi-M-560 apparatus was used to record melting points of the solid compounds; the melting points are uncorrected. IR spectra were recorded on a Perkin Elmer Spectrum One FT spectrometer. Bruker 400 and 500 MHz spectrometers were used to record NMR spectra (^{1}H, ^{13}C, APT and ^{19}F) by using TMS as the internal standard. Coupling constants (*J*) are reported in Hz. A Micromass Q-TOF mass spectrometer was used to record High Resolution Mass Spectra at 60–70 eV in ESI mode. X-ray data were collected on a Rigaku Saturn 724+ diffractometer that was equipped with a graphite monochromator using (Cu and Mo-Kα) radiation (λ = 0.71073 Å). The structures were solved by using direct methods (SHELXS97) and were refined by full-matrix least-squares against F2 by using SHELXL97 software. *N*-Tosyl and *N*-mesyl triazoles were prepared by literature methods.[1] All the 1,3-dicarbonyl compounds were commercially available (Sigma/Alfa Aesar/Spectrochem).

General procedure

N-Tosylphenyltriazole (59 mg, 0.2 mmol) and the respective1,3-dicarbonyl compound (0.2 mmol) were stirred in anhydrous chloroform (3 mL) under inert atmosphere at room temperature. After completion of the reaction (as observed by TLC) the solvent was removed and the residue was directly subjected to silica-gel column chromatography (20% EA-PE) to afford the pure product (see Scheme 2 and Scheme 3a).

Experimental data

3-(4-Phenyl-1*H*-1,2,3-triazol-1-yl)cyclohex-2-enone (3a)

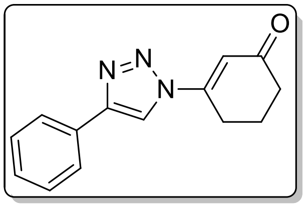

White solid; Yield 38 mg, 78%; mp 116-118 °C; IR (neat, cm^{-1}) 3137 (w), 3114 (w), 3055 (vw), 2927 (m), 2854 (w), 1668 (vs), 1622 (vs), 1455 (m), 1435 (m), 1229 (m), 1020 (m), 764 (m), 738 (s), 705 (m); ^{1}H NMR (500 MHz, $CDCl_3$) δ 2.27 (quint, *J* = 6.4 Hz, 2H), 2.57 (t, *J* = 6.4 Hz, 2H), 3.25 (t, *J* = 6.4 Hz, 2H), 6.44 (s, 1H), 7.40 (t, *J* = 7.7 Hz, 1H), 7.47 (t, *J* = 7.7 Hz, 2H),7.87 (d, *J* = 7.7 Hz, 2H), 8.12 (s, 1H); ^{13}C NMR (125 MHz, $CDCl_3$) δ 21.5, 26.0, 37.2, 115.5, 116.6, 126.2, 129.1, 129.2, 129.5, 149.0, 153.0, 198.5; HRMS (ES+) calcd for $C_{14}H_{14}N_3O$ (MH^+) 240.1131, found 240.1134.

3-(4-(4-*tert*-Butyl)phenyl-1*H*-1,2,3-triazol-1-yl)cyclohex-2-enone (3d)

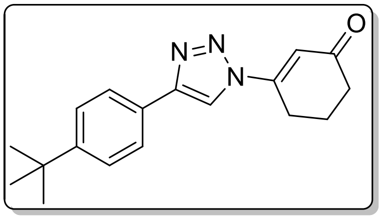

White solid; Yield 42 mg, 70%; mp 148-150 °C; IR (neat, cm^{-1}) 3121 (w), 2953 (m), 1669 (vs), 1621 (s), 1442 (s), 1228 (s); ^{1}H NMR (500 MHz, $CDCl_3$) δ 1.36 (s, 9H), 2.26 (quint, *J* = 6.5 Hz, 2H), 2.56 (t, *J* = 6.5 Hz, 2H), 3.25 (td, *J* = 6.5, 1.3 Hz, 2H), 6.43 (t, *J* = 1.3 Hz, 1H), 7.49 (d, *J* = 8.5 Hz, 2H), 7.80 (d, *J* = 8.5Hz, 2H), 8.08 (s, 1H); ^{13}C NMR (125 MHz,

$CDCl_3$) δ 21.5, 25.9, 31.4, 34.9, 37.1, 115.3, 116.2, 125.9, 126.1, 126.6, 149.0, 152.4, 153.0, 198.6; HRMS (ES+) calcd for $C_{18}H_{21}N_3OK$ (MK^+) 334.1316, found 334.1314.

3-(4-Phenyl-1*H*-1,2,3-triazol-1-yl)cyclopent-2-enone (3e)

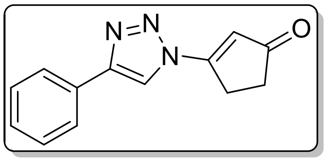

White solid; Yield 24 mg, 55%; mp 168-170 ºC; IR (neat, cm^{-1}) 3133 (vw), 2929 (w), 1664 (vs), 1622 (vs), 1579 (vs), 1462 (m), 1432 (m), 1188 (m), 1023 (m), 852 (m), 765 (s), 693 (m); ^{1}H NMR (400 MHz, $CDCl_3$) δ 2.71-2.74 (m, 2H), 3.39-3.42 (m, 2H), 6.42 (s, 1H),7.42 (t, *J* = 7.4 Hz, 1H), 7.48 (t, *J* = 7.4 Hz, 2H),7.90 (d, *J*= 7.4 Hz, 2H), 8.13 (s, 1H); ^{13}C NMR (125 MHz, $CDCl_3$) δ 27.1, 34.6, 117.5, 117.7, 126.3, 129.2, 129.3, 129.4, 149.5, 164.6, 205.0; HRMS (ES+) calcd for $C_{13}H_{12}N_3O$ (MNa^+) 226.0975, found 226.0975; Selected X-ray data (CCDC 2044636): $C_{13}H_{11}N_3O$, *M* = 225.25, Monoclinic, space group *P2₁/n*, a = 5.890, Å, *b* = 24.263 , Å, *c* = 7.661, Å, *α* = 90°, *β* = 99.67°, *γ* = 90°, *V* = 1079.3 $Å^3$, D_x = 1.386 Mg m^{-3}, *Z* = 4, F(000) = 472.0, λ =0.71073Å, μ = 0.092 cm-^{1}, total/unique= 1881/1881[R(int) = 0.0876], T=293(2) K, θ range = $θ_{max}$ = 24.996°, $θ_{min}$ = 3.117°, final R[I>2σ(I)]: *R1* = R_1 = 0.0511, wR_2 = 0.1082, *R(all data)*: R_1 = 0.1056, wR_2 = 0.1493.

3-(4-(p-Tolyl-1*H*-1,2,3-triazol-1-yl)cyclopent-2-enone (3f)

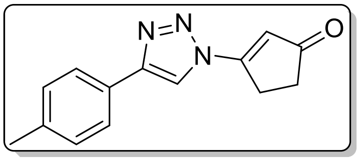

White solid; Yield 34 mg, 71%; mp 183-185 ºC; IR (neat, cm^{-1}) 2922 (w), 2854 (vw), 1717 (s), 1697 (m), 1621 (m), 1219 (w), 1049 (m), 750 (vs); ^{1}H NMR (400 MHz, $CDCl_3$) δ 2.41 (s, 3H), 2.70-2.73 (m, 2H), 3.39-3.41 (m, 2H), 6.40 (s, 1H), 7.28 (d, *J* = 7.9 Hz, 2H), 7.79 (d, *J* = 7.9 Hz, 2H), 8.08 (s, 1H); ^{13}C NMR (125 MHz, $CDCl_3$) δ 21.5, 27.1, 34.6, 117.3, 117.4, 126.2, 126.3, 129.9, 139.4, 149.5, 164.6, 205.0; HRMS (ES+) calcd for $C_{14}H_{13}NONa$ ($[M-N_2]Na^+$) 234.0889, found 234.0884.

3-(4-Methoxyphenyl-1*H*-1,2,3-triazol-1-yl)cyclopent-2-enone (3g)

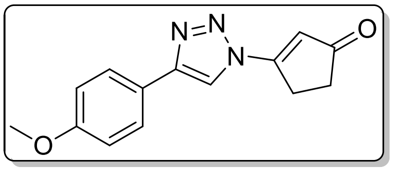

Whitesolid; Yield 32 mg, 61%; mp 168-170 ºC; IR (neat, cm^{-1}) 2922 (m), 2855 (w), 1716 (w), 1624 (vvs), 1262 (w), 1030 (m), 849 (w), 765 (w); ^{1}H NMR (500 MHz, $CDCl_3$) δ 2.70-2.72 (m, 2H), 3.38-3.40 (m, 2H), 3.86 (s, 3H), 6.39 (s, 1H),6.99 (d, *J* = 8.8 Hz, 2H), 7.82 (d, *J* = 8.8 Hz, 2H), 8.03 (s, 1H); ^{13}C NMR (125 MHz, $CDCl_3$) δ 27.1, 34.6, 55.6, 114.7, 116.7, 117.3, 121.8, 127.7, 149.4, 160.6, 164.7, 205.1; HRMS (ES+) calcd for $C_{14}H_{14}NO_2$ ($[M-N_2]H^+$) 228.1019, found 228.1015.

3-(3-Methoxyphenyl-1*H*-1,2,3-triazol-1-yl)cyclopent-2-enone (3h)

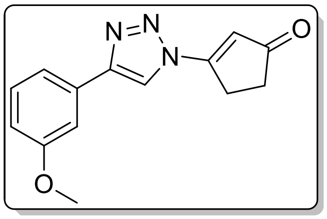

White solid; Yield 20 mg, 38%; mp 168-170 ºC; IR (neat, cm^{-1}) 3131 (w), 2931(w), 1710 (vs), 1623 (s), 1584 (m), 1436 (m), 1256 (m), 1044 (m), 1017 (s), 787 (m); ^{1}H NMR (500 MHz, $CDCl_3$) δ 2.70-2.72 (m, 2H), 3.37-3.39 (m, 2H), 3.88 (s, 3H), 6.42 (s, 1H), 6.95 (dd, *J* = 7.9, 2.1 Hz, 1H), 7.37 (t, *J* = 7.9 Hz, 1H), 7.42 (d, *J* = 7.9 Hz, 1H), 7.40 (d, *J* = 2.1 Hz, 1H), 8.12 (s, 1H);^{13}C NMR (125 MHz, $CDCl_3$) δ 27.1, 34.6, 55.6, 111.5, 115.2, 117.6,

117.9, 118.6, 130.3, 130.4, 149.3, 160.3, 164.5, 205.0; HRMS (ES+) calcd for $C_{14}H_{14}N_3O_2$ (MH^+) 256.1081, found 256.1085.

3-(4-(4-*tert*-Butyl)phenyl-1*H*-1,2,3-triazol-1-yl)cyclopent-2-enone (3i)

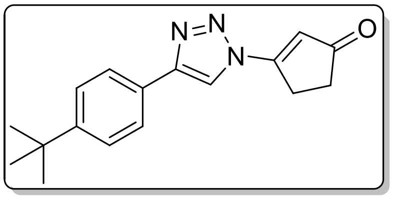

White solid; Yield 30 mg, 53%; mp 233-235 °C; IR (neat, cm^{-1}) 3115 (m), 3086 (w), 2959 (s), 1715 (s), 1697 (m), 1621 (vs), 1434 (s), 1028 (m), 809 (w); ^{1}H NMR (500 MHz, $CDCl_3$) δ 1.36 (s, 9H) 2.70-2.72 (m, 2H), 3.39-3.41 (m, 2H), 6.41 (s, 1H), 7.50 (d, *J* = 8.3 Hz, 2H), 7.82 (d, *J* = 8.3 Hz, 2H), 8.09 (s, 1H); ^{13}C NMR (125 MHz, $CDCl_3$) δ 27.1, 31.4, 34.6, 35.0, 117.3, 117.4, 126.1, 126.2, 126.3, 149.5, 152.7, 164.6, 205.1; HRMS (ES+) calcd for $C_{17}H_{19}NONa$ ($[M\text{-}N_2]Na^+$) 276.1359, found 276.1358.

3-(4-(4-Fluorophenyl)-1*H*-1,2,3-triazol-1-yl)cyclopent-2-enone (3j)

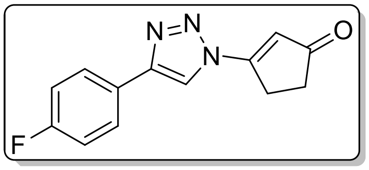

White solid; Yield 32 mg, 67%; mp 173-175 °C; IR (neat, cm^{-1}) 3114 (w), 1713 (vs), 1698 (vs), 1620 (vs), 1459 (m), 1444 (m), 1239 (s), 1218 (s), 859 (s), 838 (s), 807 (s); ^{1}H NMR (400 MHz, $CDCl_3$) δ 2.69-2.72 (m, 2H), 3.37-3.39 (m, 2H), 6.41 (s, 1H), 7.15 (t, *J* = 8.6 Hz, 2H), 7.86 (dd, *J* = 8.6, 5.3 Hz, 2H), 8.12 (s, 1H); ^{13}C NMR (125 MHz, $CDCl_3$) δ 27.1, 34.6, 116.3 (d, *J* = 22.5 Hz), 117.5, 117.6, 125.4, 128.1 (d, *J* = 7.5 Hz), 148.5, 163.4 (d, *J* = 248.8 Hz), 164.5, 205.0; ^{19}F NMR (470 MHz, $CDCl_3$) δ -111.7; HRMS (ES+) calcd for $C_{13}H_{11}FN_3O$ (MH^+) 244.0881, found 244.0883.

3-(4-(4-Chlorophenyl)-1*H*-1,2,3-triazol-1-yl)cyclopent-2-enone (3k)

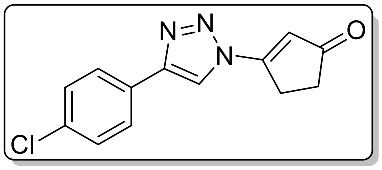

White solid; Yield 38 mg, 52%; mp 184-186 °C; IR (neat, cm^{-1}) 2933 (w), 1713 (m), 1689 (w), 1616 (vs), 1455 (m), 1431 (m), 1236 (m), 1216 (m), 1022 (s), 812 (m), 742 (vs); ^{1}H NMR (500 MHz, $CDCl_3$) δ 2.71-2.73 (m, 2H), 3.38-3.40 (m, 2H), 6.42 (t, *J* = 1.6 Hz, 1H), 7.45 (d, *J* = 8.5 Hz, 2H), 7.83 (d, *J* = 8.5 Hz, 2H), 8.12 (s, 1H); ^{13}C NMR (125 MHz, $CDCl_3$) δ 27.1, 34.6, 117.7, 117.8, 127.5, 127.7, 129.5, 135.3, 148.4, 164.4, 204.9; HRMS (ES+) calcd for $C_{13}H_{10}ClN_3ONa$ (MNa^+) 282.0405, found 282.0399.

3-(4-(4-Bromophenyl)-1*H*-1,2,3-triazol-1-yl)cyclopent-2-enone (3l)

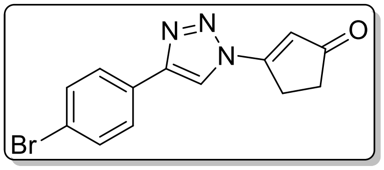

White solid; Yield 34mg, 54%; mp 205-207 °C; IR (neat, cm^{-1}) 2930 (vw), 1708 (vvs), 1693 (vvs), 1622 (vs), 1451 (vs), 1430 (vs), 1237 (m), 1215 (m), 1184 (m), 1072 (vw), 1024 (s), 1015 (s), 807 (m); ^{1}H NMR (500 MHz, DMSO-d_6) δ 2.59-2.61 (unresolved m, 2H), 3.26-3.28 (unresolved m, 2H), 6.61 (s, 1H), 7.70 (d, *J* = 6.4 Hz, 2H), 7.85 (d, *J* = 6.4 Hz, 2H), 9.31 (s, 1H); ^{13}C NMR (125 MHz, DMSO) δ 26.5, 34.3, 117.4, 120.8, 121.8, 127.5, 128.6, 132.1, 146.7, 164.9, 205.2; HRMS (ES+) calcd for $C_{13}H_{11}BrN_3O$ (MH^+) 304.0080, found 304.0083.

3-(4-(Thiophen-2-yl)-1*H*-1,2,3-triazol-1-yl)cyclopent-2-enone (3m)

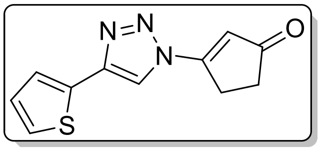

White solid; Yield 34 mg, 75%; mp 205-207 °C; IR (neat, cm^{-1}) 3150 (w), 3044 (w), 2936 (w), 1718 (s), 1692 (vs), 1618 (vs), 1454 (s), 1430 (s), 1265 (m), 1205 (m), 1183 (m), 1040 (s), 860 (m), 801 (s), 743 (vvs); ^{1}H NMR (500 MHz, DMSO-d_6) δ 1.74-1.76 (m, 2H), 2.43-2.45 (m, 2H), 5.80 (s, 1H), 6.36 (dd, *J* = 4.5, 3.0 Hz, 1H), 6.70 (d, *J* = 3.0 Hz, 1H), 6.81 (d, *J* = 4.5 Hz, 1H), 8.36 (s, 1H); ^{13}C NMR (125 MHz, DMSO) δ 26.5, 34.3, 117.4, 119.5, 125.3, 126.7, 128.2, 131.3, 143.2, 164.9, 205.3; HRMS (ES+) calcd for $C_{11}H_{10}N_3OS$ (MH^+) 232.0539.

(1-(3-Oxocyclopent-1-en-1-yl)-1*H*-1,2,3-triazol-4-yl)methyl benzoate (3n)

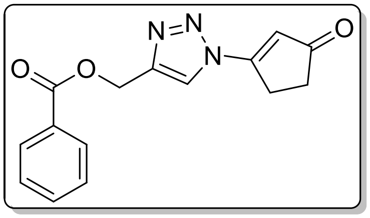

White solid; Yield 46 mg, 82%; mp 128-130 °C; IR (neat, cm^{-1}) 3107 (w), 3085 (w), 2929 (w), 1709 (vs), 1620 (m), 1599 (m), 1453 (m), 1267 (s), 1243 (m), 716 (s); ^{1}H NMR (500 MHz, $CDCl_3$) δ 2.68-2.70 (m, 2H), 3.32-3.35 (m, 2H), 5.54 (s, 2H), 6.42 (t, *J* = 1.6 Hz, 1H), 7.44 (t, *J* = 7.8 Hz, 2H), 7.58 (t, *J* = 7.8 Hz, 1H), 8.05 (d, *J* = 7.8 Hz, 2H), 8.09 (s, 1H); ^{13}C NMR (125 MHz, $CDCl_3$) δ 27.1, 34.6, 57.7, 118.1, 122.9, 128.7, 129.5, 129.9, 133.6, 144.8, 164.3, 166.6, 204.8; HRMS (ES+) calcd for $C_{15}H_{13}N_3O_3Na$ (MNa^+) 306.0849, found 306.0849.

3-(4-Propyl-1*H*-1,2,3-triazol-1-yl)cyclopent-2-enone (3o)

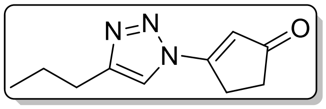

White solid; Yield 20 mg, 52%; mp 101-103 °C; IR (neat, cm^{-1}) 3128 (w), 3077 (w), 2959 (m), 2934 (m), 2874 (w), 1714 (vs), 1698 (vs), 1621 (vs), 1457 (s), 1431 (m), 1189 (s), 1045 (s), 868 (s); ^{1}H NMR (500 MHz, $CDCl_3$) δ 1.00 (t, *J* = 7.5 Hz, 3H), 1.74 (sext, *J* = 7.5 Hz, 2H), 2.66-2.68 (m, 2H), 2.76 (t, *J* = 7.5 Hz, 2H), 3.32-3.35 (m, 2H), 6.31 (d, *J* = 1.3Hz, 1H), 7.67 (s, 1H); ^{13}C NMR (125 MHz, $CDCl_3$) δ 13.9, 22.5, 27.0, 27.6, 34.5, 116.9, 119.4, 150.3, 164.9, 205.2; HRMS (ES+) calcd for $C_{10}H_{14}N_3O$ (MH^+) 192.1131, found 192.1129.

2- Methyl-3-(4-phenyl-1*H*-1,2,3-triazol-1-yl)cyclopent-2-enone (3p)

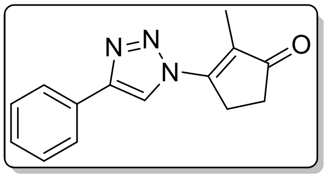

White solid; Yield 26 mg, 55%; mp 179-180 °C; IR (neat, cm^{-1}) 3140 (m), 2929 (m), 1703 (vs), 1645 (vvs), 1455 (m), 1436 (s), 1301 (m), 1240 (m), 1012 (m), 767 (vs), 696 (m); ^{1}H NMR (400 MHz, $CDCl_3$) δ 2.18 (t, *J* = 2.0 Hz, 3H), 2.69-2.72 (m, 2H), 3.23-3.28 (m, 2H), 7.40 (t, *J* = 7.2 Hz, 1H), 7.48 (t, *J* = 7.2 Hz, 2H), 7.91 (d, *J* = 7.2 Hz, 2H),8.16 (s, 1H); ^{13}C NMR (125 MHz, $CDCl_3$) δ 9.7, 26.6, 33.4, 118.2, 126.1, 127.3, 129.1, 129.2, 129.4, 148.5, 157.1, 206.0; HRMS (ES+) calcd for $C_{14}H_{14}N_3O$ (MH^+) 240.1131, found 240.1130.

5,5-Dimethyl-3-oxocyclohex-1-en-yl methanesulfonate (4a) (Scheme 3a)

N-Mesyl phenyl triazole (**1a'**, 44 mg, 0.2 mmol, 1 equiv) and dimedone (**2d**, 28 mg, 0.2 mmol, 1 equiv) were stirred in anhydrous chloroform (3 mL) under inert atmosphere at room temperature for 48 h. After

completion of reaction, the solvent was removed in vacuo and the residue was directly subjected to silica-gel column chromatography (15% EA-PE) to afford the pure product **4a**.

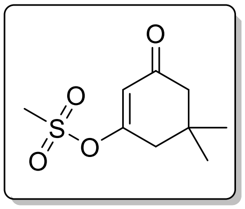

Colourless liquid; Yield 20 mg, 45%; IR (neat, cm^{-1}) 3019 (w), 2962 (vs), 2873 (m), 1666 (vs), 1614 (vs), 1469 (m), 1371 (vs), 1355 (vs), 1095 (m), 734 (m), 538 (m); ^{1}H NMR (500 MHz, $CDCl_3$) δ 2.08 (s, 6H), 2.25 (s, 2H), 3.45 (d, *J* = 0.9 Hz, 2H), 3.19 (s, 3H), 6.02 (br s, 1H); ^{13}C NMR (100 MHz, $CDCl_3$) δ 32.4, 32.6, 38.6, 41.2, 41.9, 50.1, 114.7, 166.1, 198.3; HRMS (ES+) calcd for $C_9H_{14}O_4S$ (MH^+) 219.0686, found 219.0684.

Control experiment (Scheme 3b)

N-Tosylphenyltriazole **1a** (59 mg, 0.2 mmol, 1 equiv) and cyclopentane-1,3-dione (**2b**, 20 mg, 0.2 mmol, 1 equiv) were stirred in anhydrous chloroform (3 mL) under inert atmosphere at room temperature. The reaction was stopped after 10 h before the completion of the reaction. The solvent was removed in vacuo and the residue was directly subjected to silica-gel column chromatography (20% EA-PE) to afford the products **3e** as a white solid in 40% yield (18 mg) and **4b'** as a colourless liquid in 30% yield (15 mg).

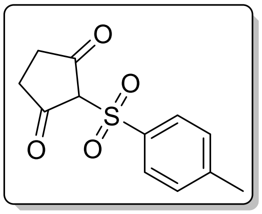

2-Tosylcyclopentane-1,3-dione (4b'): Colorless liquid; Yield 15 mg, 30%; IR (neat, cm^{-1}) 3428 (br m), 2929 (w), 1661 (w), 1567 (m), 1426 (m), 1172 (vs), 1124 (vs), 1034 (s), 1010 (s), 842 (m), 818 (m), 686 (s), 568 (s); ^{1}H NMR (400 MHz, DMSO-d_6) δ 2.28 (s, 3H), 2.37 (s, 4H), 5.10 (s, 1H), 7.14 (d, *J* = 8.0 Hz, 2H), 7.50 (d, *J* = 8.0 Hz, 2H); ^{13}C NMR (100 MHz, DMSO-d_6) δ 21.1, 31.6, 105.3, 125.8, 128.7, 138.9, 144.6, 198.8; HRMS (ES+) calcd for $C_{12}H_{12}O_4S$ (MH^+) 252.0451, found 252.0452.

Rearrangement of 4b to 4b' (Scheme 3c)

3-Oxocyclopent-1-en-1-yl-4-methylbenzenesulfonate (**4b**)[2] (50 mg, 0.2 mmol, 1 equiv) was kept at room temperature overnight (12 h) which completely converted to **4b'** (colorless liquid) in quantitative yield.

3-(Benzylamino)cyclopent-2-en-1-one (6b, Scheme 3e)

To a stirred solution of benzyl amine (**5a**, 72 mg, 0.2 mmol, 1 equiv) in chloroform (3 mL), the 3-oxocyclopent-1-en-1-yl-4-methylbenzenesulfonate (**4b**, 50 mg, 0.2 mmol, 1 equiv) was added and the stirring was continued for 48 h at room temperature. After completion of the reaction (monitored by TLC), the solvent was removed in vacuo and the residue was directly subjected to silica-gel column chromatography (20% Ethyl acetate/MeOH) to afford the pure product **6b**.

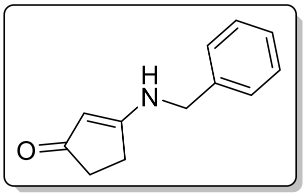

White solid; Yield 27 mg, 72%; mp 121-123 °C; IR (neat, cm^{-1}) 3227 (br s), 3058 (m), 2920 (w), 1645 (m), 1563 (vs), 1435 (m), 1274 (m), 1194 (s), 1067 (m), 749 (m), 702 (m), 631 (m); ^{1}H NMR (500 MHz, $CDCl_3$) δ 2.42 (poorly resolved t, *J* = 5.2 Hz, 2H), 2.60 (poorly resolved t, *J* = 5.2 Hz, 2H), 4.31 (d, *J* = 4.1 Hz, 2H), 5.15 (s, 1H), 5.25 (br s, 1H), 7.26-7.38 (m, 5H); ^{13}C NMR

(100 MHz, DMSO-d_6) δ 27.4, 33.6, 47.9, 97.9, 127.3, 127.5, 128.6, 138.2, 177.1, 202.8; HRMS (ES+) calcd for $C_{12}H_{13}NNaO$ (MNa^+) 210.0889 found 210.0885.

References

1. Raushel, J.; Fokin, V. V. *Org. Lett*. **2010**, *12*, 4952-4955. doi.org/10.1021/ol102087r
2. Kremsmair, A.; Skotnitzki, J.; Knochel, P. *Chem. – Eur. J.* **2020,** *26*, 11971–11973. doi.org/10.1002/chem.202002297
