# Supplementary material for: Synthesis of β-triazolylenones via metal-free desulfonylative alkylation of N-tosyl-1,2,3-triazoles
Source: Beilstein J Org Chem. 2021 Mar 31;17:762–70. doi: 10.3762/bjoc.17.66 (PMC8022205; doi:10.3762/bjoc.17.66)
Supplement: File 2 — Copies of NMR spectra. [file Beilstein_J_Org_Chem-17-762-s002.pdf]

BEILSTEIN JOURNAL OF ORGANIC CHEMISTRY

Supporting Information

for

Synthesis of β-triazolylenones via metal-free desulfonylative alkylation of *N*-tosyl-1,2,3-triazoles

Soumyaranjan Pati, Renata G. Almeida, Eufrânio N. da Silva Júnior
and Irishi N. N. Namboothiri

Beilstein J. Org. Chem. **2021,** *17,* 762–770. doi:10.3762/bjoc.17.66

Copies of NMR spectra

License and Terms: This is a supporting information file under the terms of the Creative Commons Attribution License (https://creativecommons.org/licenses/by/4.0). Please note that the reuse, redistribution and reproduction in particular requires that the author(s) and source are credited and that individual graphics may be subject to special legal provisions.
The license is subject to the *Beilstein Journal of Organic Chemistry* terms and conditions: (https://www.beilstein-journals.org/bjoc/terms)

Entry	Table of contents	Page
01	**Figure S01: ^{1}H NMR spectrum of 3a**	S03
02	**Figure S02: ^{13}C NMR spectrum of 3a**	S04
03	**Figure S03: ^{1}H NMR spectrum of 3d**	S05
04	**Figure S04: ^{13}C NMR spectrum of 3d**	S06
05	**Figure S05: ^{1}H NMR spectrum of 3e**	S07
06	**Figure S06: ^{13}C NMR spectrum of 3e**	S08
07	**Figure S07: ^{1}H NMR spectrum of 3f**	S09
08	**Figure S08: ^{13}C NMR spectrum of 3f**	S10
09	**Figure S09: ^{1}H NMR spectrum of 3g**	S11
10	**Figure S10: ^{13}C NMR spectrum of 3g**	S12
11	**Figure S11: ^{1}H NMR spectrum of 3h**	S13
12	**Figure S12: ^{13}C NMR spectrum of 3h**	S14
13	**Figure S13: ^{1}H NMR spectrum of 3i**	S15
14	**Figure S14: ^{13}C NMR spectrum of 3i**	S16
15	**Figure S15: ^{1}H NMR spectrum of 3j**	S17
16	**Figure S16: ^{13}C NMR spectrum of 3j**	S18
17	**Figure S17: ^{13}C-APT NMR spectrum of 3j**	S19
18	**Figure S18: ^{19}F NMR spectrum of 3j**	S20
19	**Figure S19: ^{1}H NMR spectrum of 3k**	S21
20	**Figure S20: ^{13}C NMR spectrum of 3k**	S22
21	**Figure S21: ^{1}H NMR spectrum of 3l**	S23
22	**Figure S22: ^{13}C NMR spectrum of 3l**	S24
23	**Figure S23: ^{1}H NMR spectrum of 3m**	S25
24	**Figure S24: ^{13}C NMR spectrum of 3m**	S26
25	**Figure S25: ^{1}H NMR spectrum of 3n**	S27
26	**Figure S26: ^{13}C NMR spectrum of 3n**	S28
27	**Figure S27: ^{1}H NMR spectrum of 3o**	S29
28	**Figure S28: ^{13}C NMR spectrum of 3o**	S30
29	**Figure S29: ^{1}H NMR spectrum of 3p**	S31
30	**Figure S30: ^{13}C NMR spectrum of 3p**	S32

31	**Figure S31: ^{13}C-APT NMR spectrum of 3p**	S33
32	**Figure S32: ^{1}H NMR spectrum of 4a**	S34
33	**Figure S33: ^{13}C NMR spectrum of 4a**	S35
34	**Figure S34: ^{1}H NMR spectrum of 4b'**	S36
35	**Figure S35: ^{13}C NMR spectrum of 4b'**	S37
36	**Figure S36: ^{13}C-APT NMR spectrum of 4b'**	S38
37	**Figure S37: ^{1}H NMR spectrum of 6b**	S39
38	**Figure S38: ^{13}C NMR spectrum of 6b**	S40
39	**Figure S39: ^{13}C-APT NMR spectrum of 6b**	S41
40	**Figure S40: ORTEP diagram of 3e**	S42
41	**Table S1: Crystal table of 3e**	S42

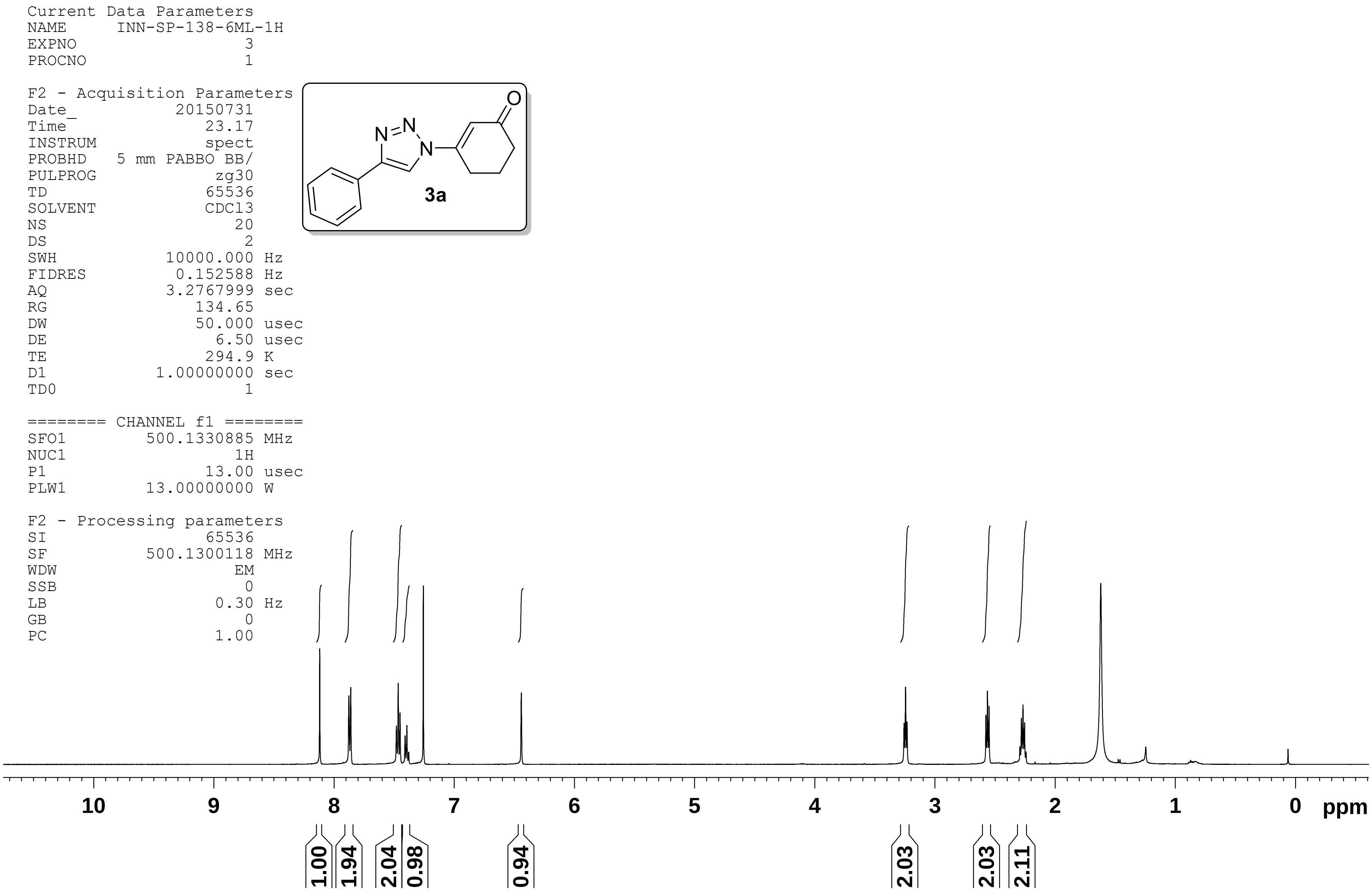

Figure S01: ^{1}H NMR spectrum of 3a.

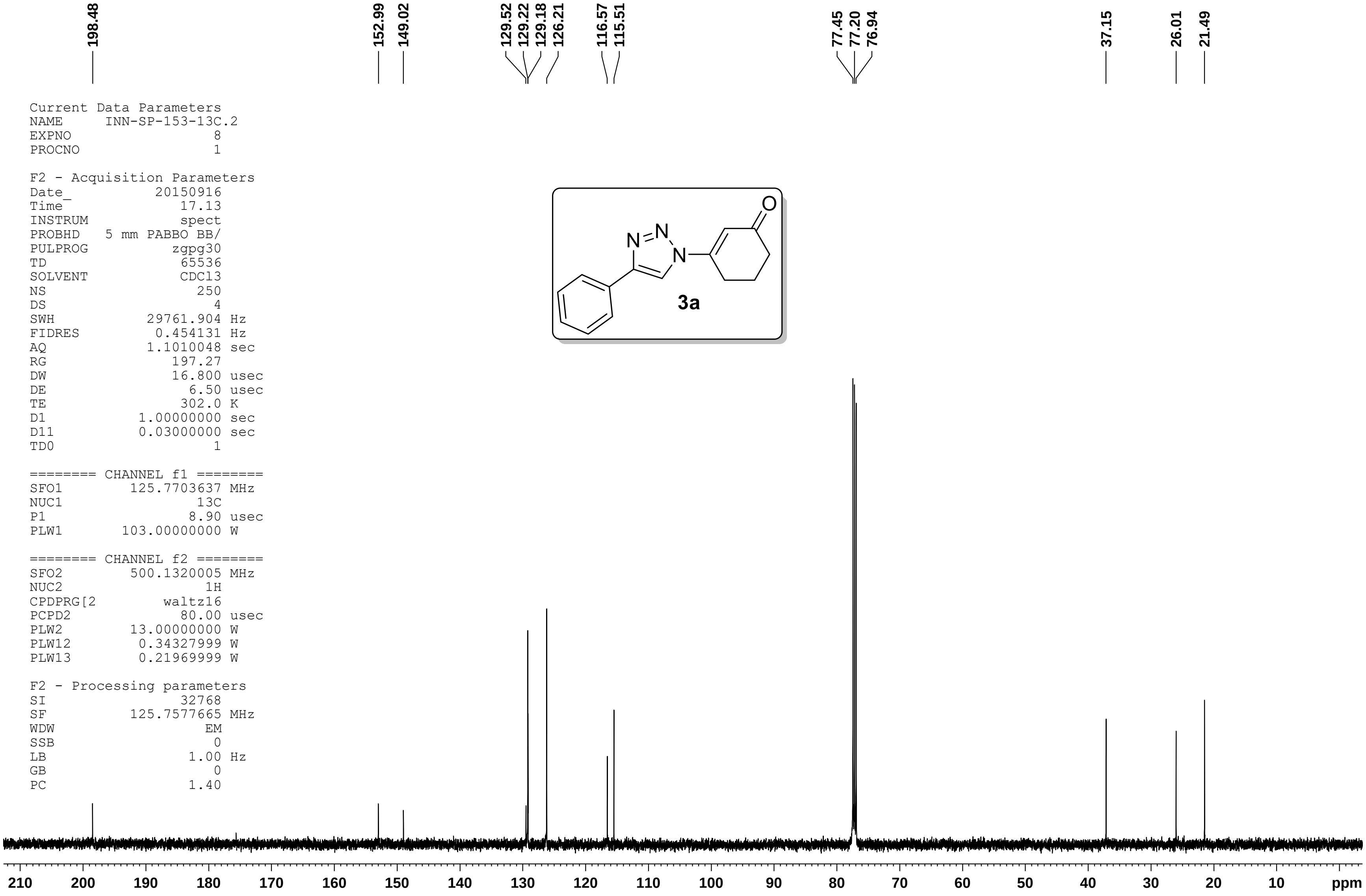

Figure S02: ^{13}C NMR spectrum of 3a.

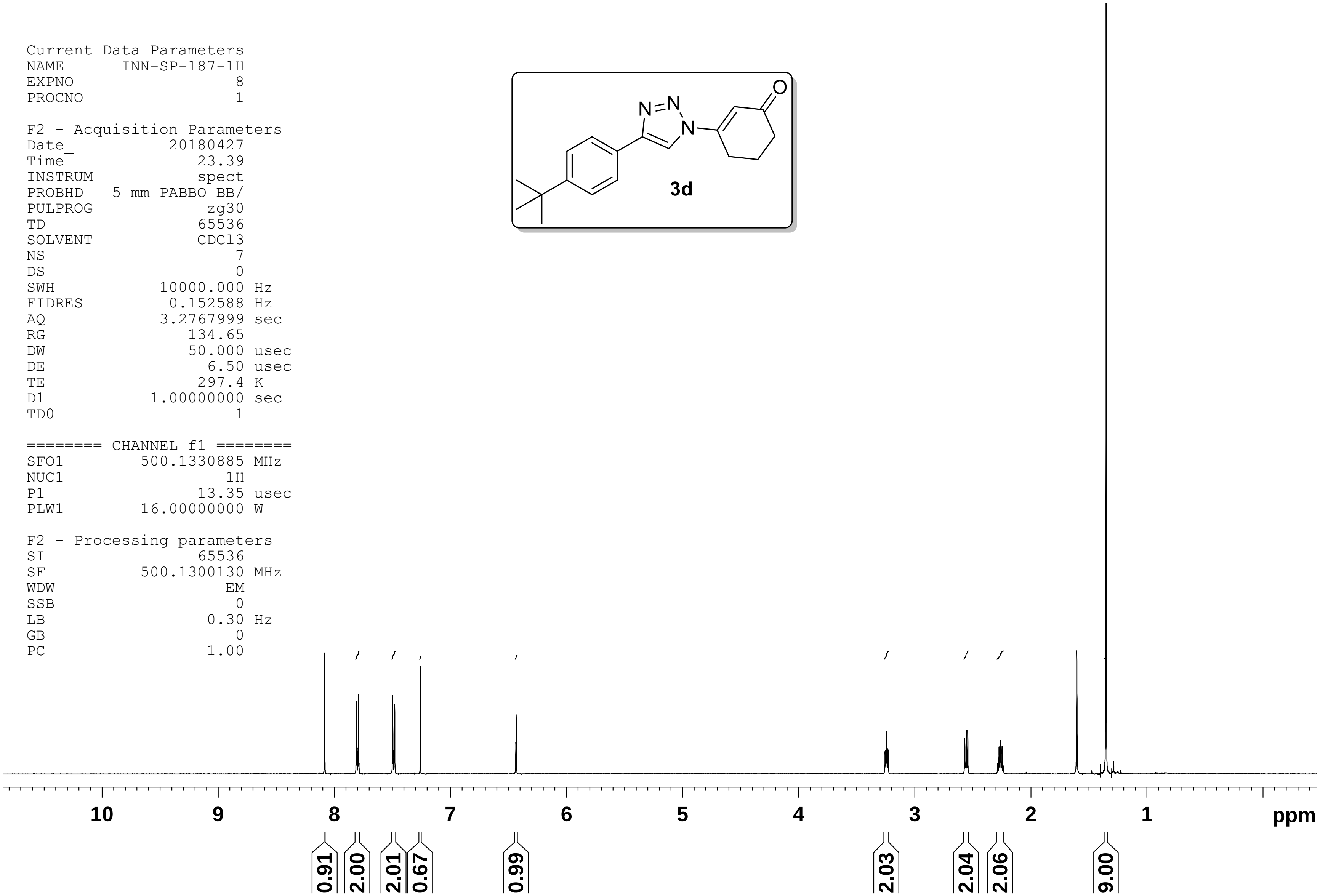

Figure S03: ^{1}H NMR spectrum of 3d.

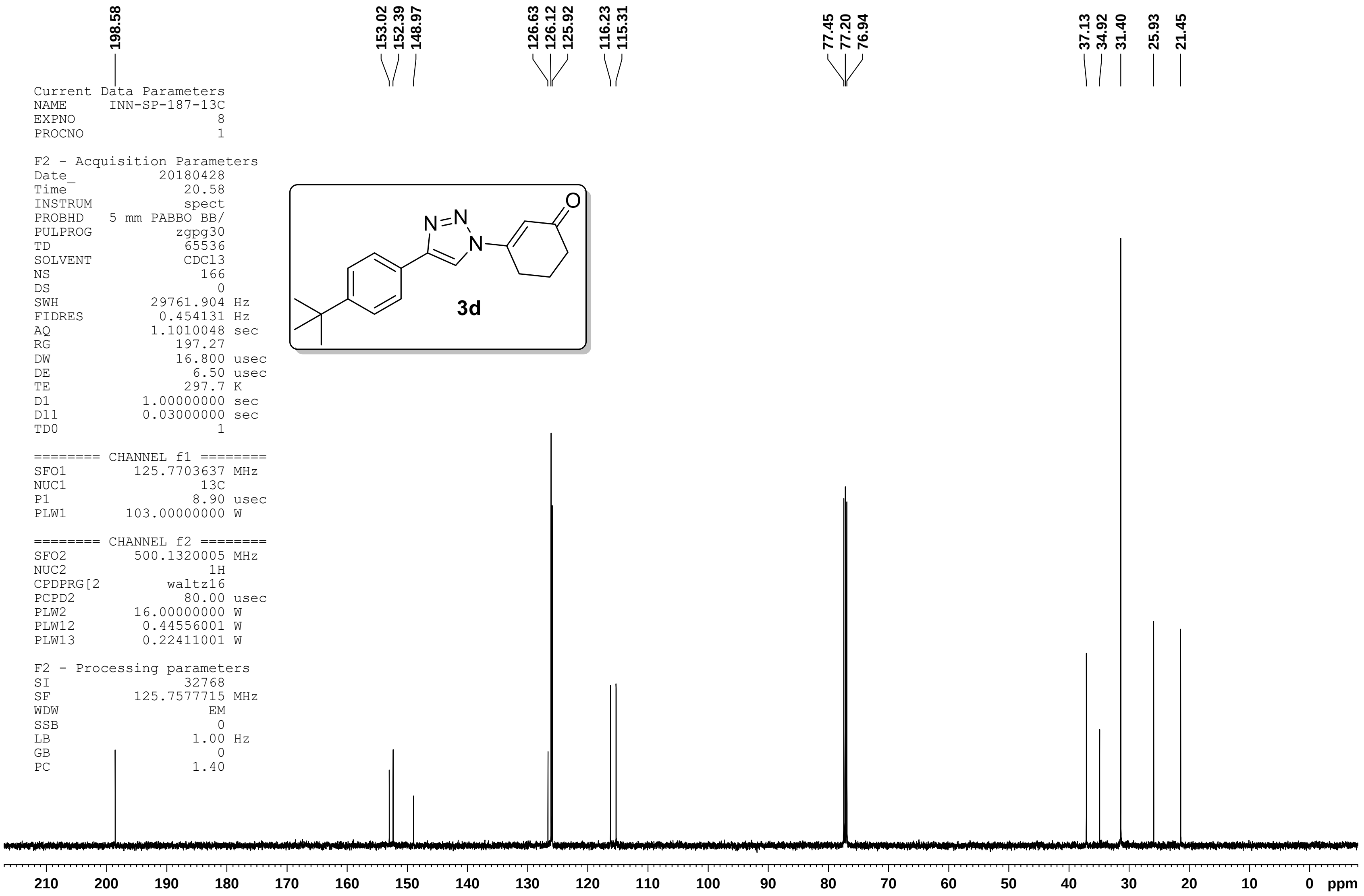

Figure S04: ^{13}C NMR spectrum of 3d.

```
Current Data Parameters
NAME      INN-SP-161-1H
EXPNO                71
PROCNO                1

F2 - Acquisition Parameters
Date_          20151011
Time               7.43
INSTRUM           spect
PROBHD   5 mm PABBO BB-
PULPROG            zg30
TD                54274
SOLVENT           CDCl3
NS                   16
DS                    0
SWH            8223.685 Hz
FIDRES         0.151522 Hz
AQ            3.2998593 sec
RG                  228
DW               60.800 usec
DE                 6.50 usec
TE                295.3 K
D1           1.00000000 sec
TD0                   1

======== CHANNEL f1 ========
NUC1                 1H
P1                14.75 usec
PL1               -1.00 dB
PL1W        10.56200695 W
SFO1        400.1324710 MHz

F2 - Processing parameters
SI                32768
SF          400.1300099 MHz
WDW                  EM
SSB                   0
LB                 0.30 Hz
GB                    0
PC                 1.00
```

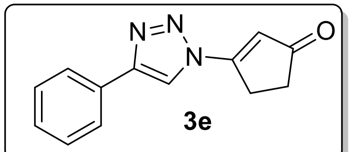

3e

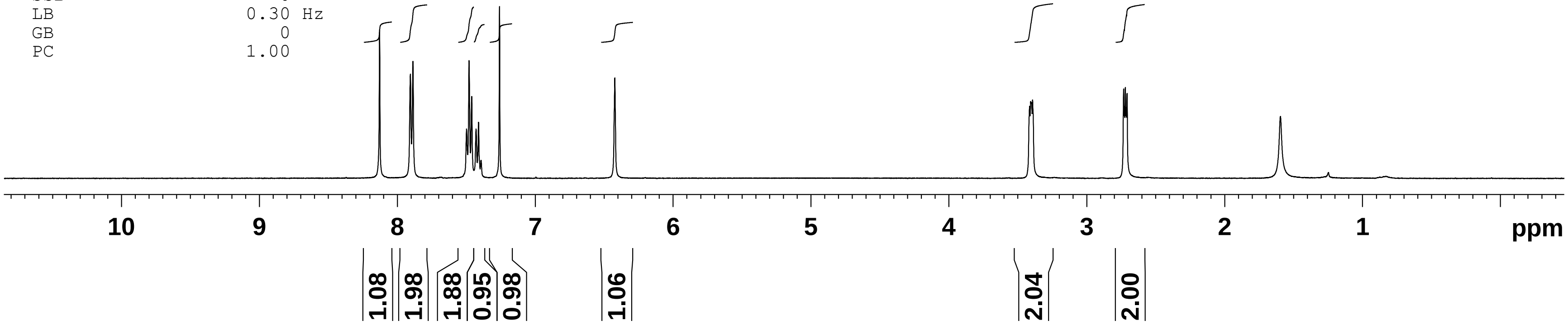

Figure S05: ^{1}H NMR spectrum of 3e.

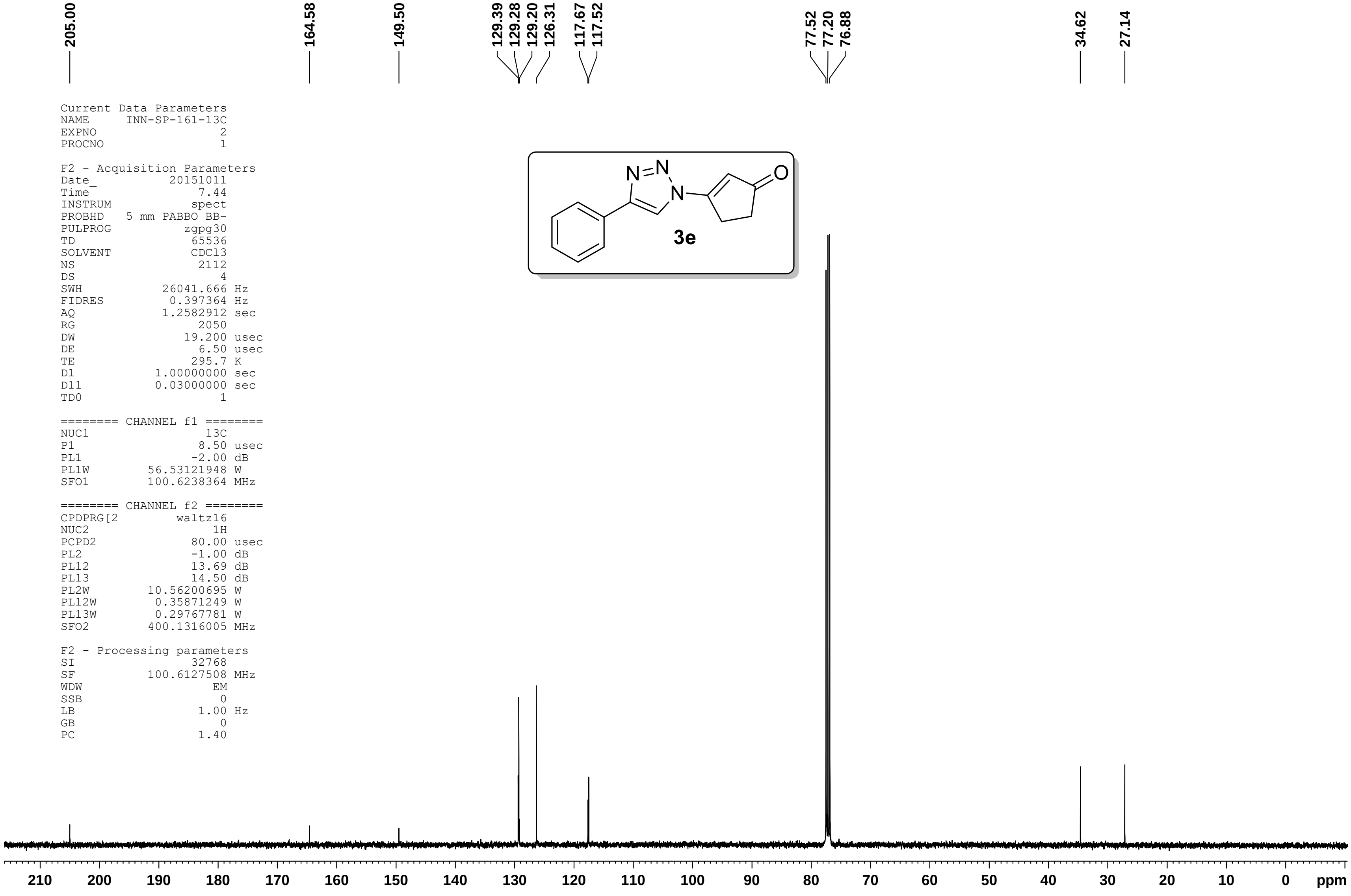

Figure S06: ^{13}C NMR spectrum of 3e.

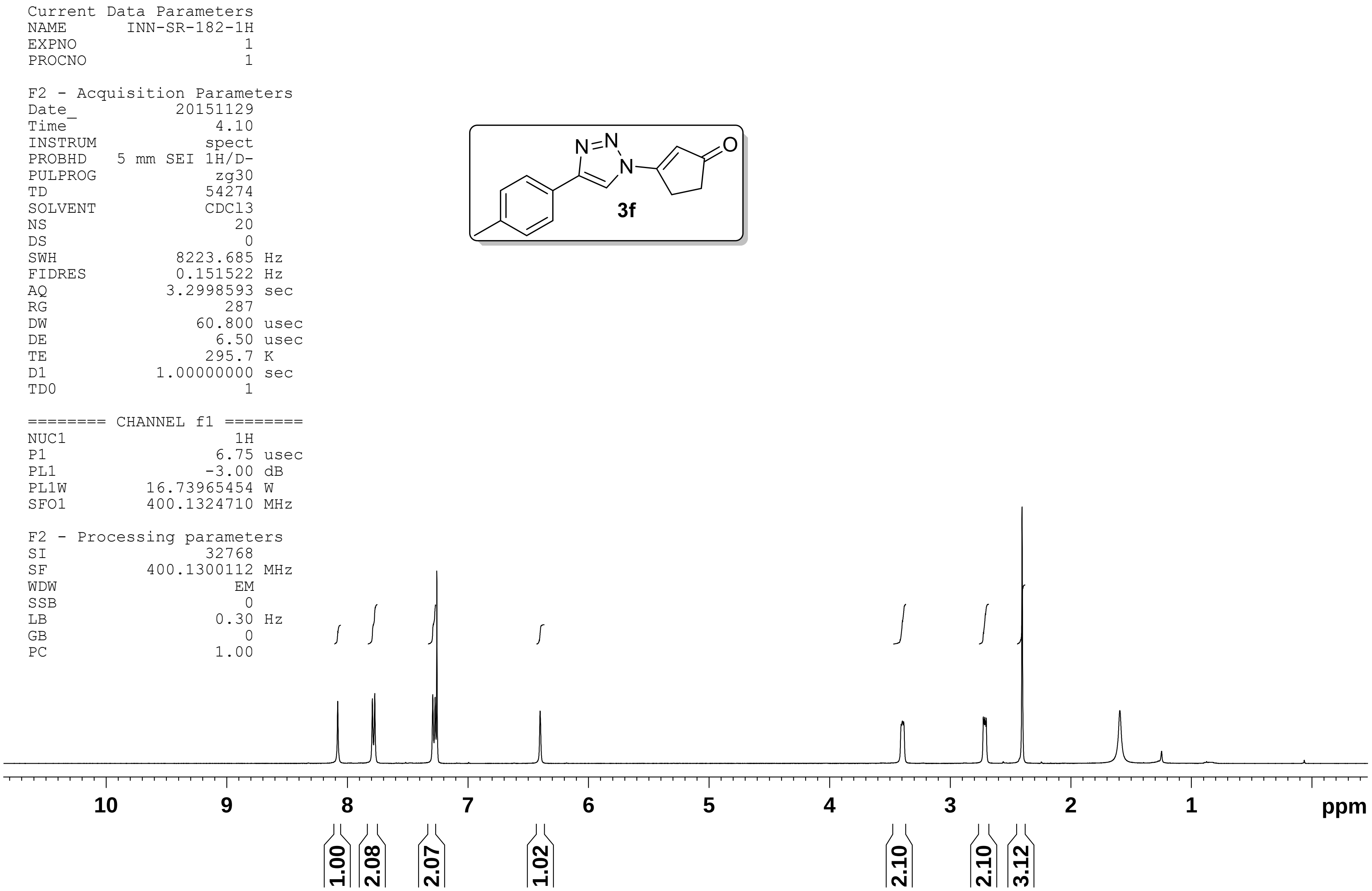

Figure S07: ^{1}H NMR spectrum of 3f.

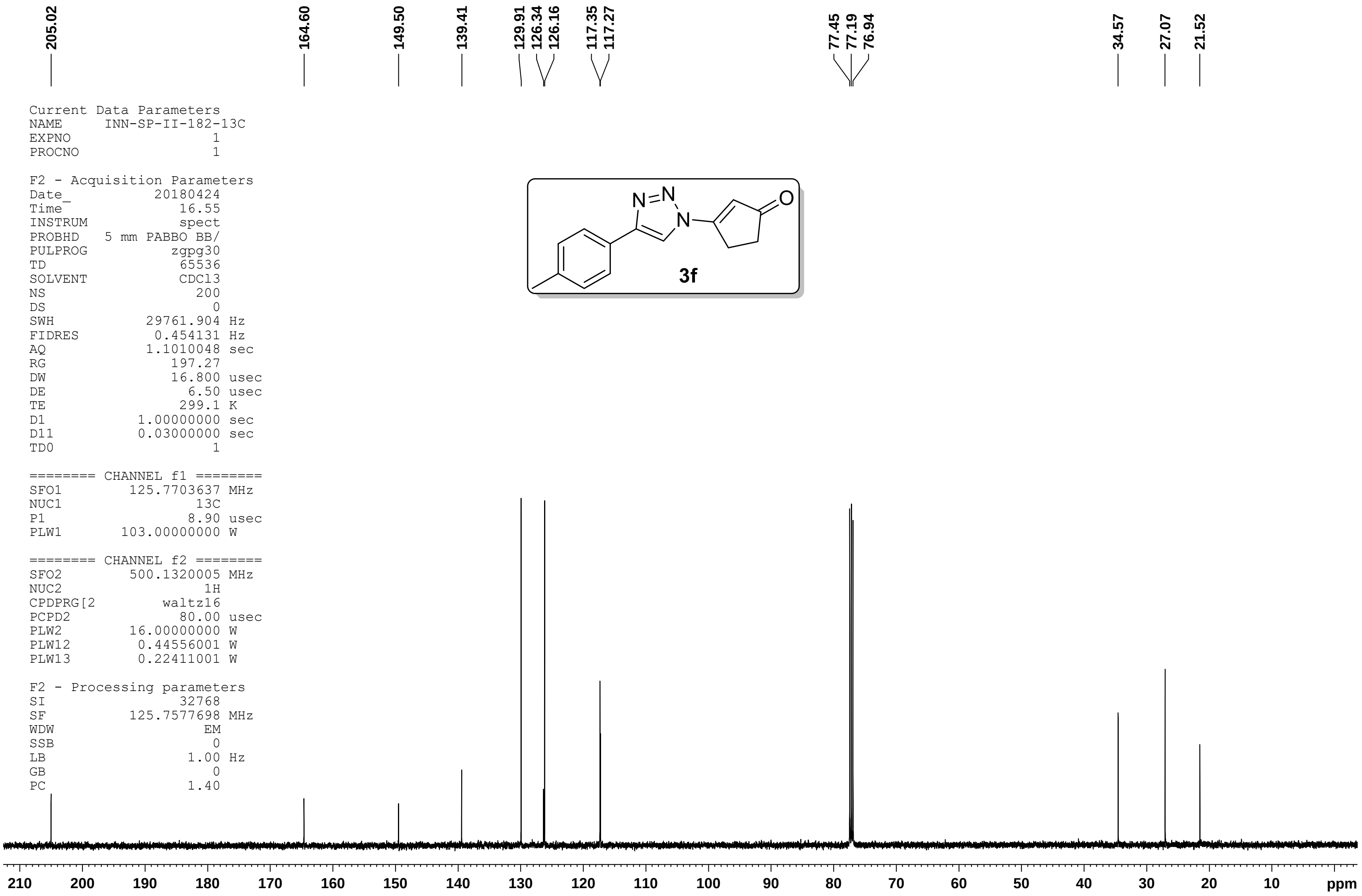

Figure S08: ^{13}C NMR spectrum of 3f.

```
Current Data Parameters
NAME      INN-SP-184-1H
EXPNO                 1
PROCNO                1

F2 - Acquisition Parameters
Date_          20151203
Time              18.36
INSTRUM           spect
PROBHD   5 mm PABBO BB/
PULPROG            zg30
TD                65536
SOLVENT           CDCl3
NS                   11
DS                    2
SWH           10000.000 Hz
FIDRES         0.152588 Hz
AQ            3.2767999 sec
RG               134.65
DW               50.000 usec
DE                 6.50 usec
TE                296.9 K
D1           1.00000000 sec
TD0                   1

======== CHANNEL f1 ========
SFO1        500.1330885 MHz
NUC1                 1H
P1                13.00 usec
PLW1        13.00000000 W

F2 - Processing parameters
SI                65536
SF          500.1300124 MHz
WDW                  EM
SSB                   0
LB                 0.30 Hz
GB                    0
PC                 1.00
```

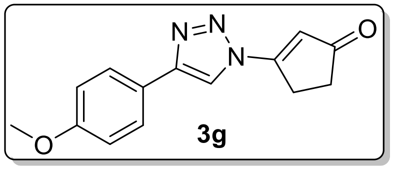

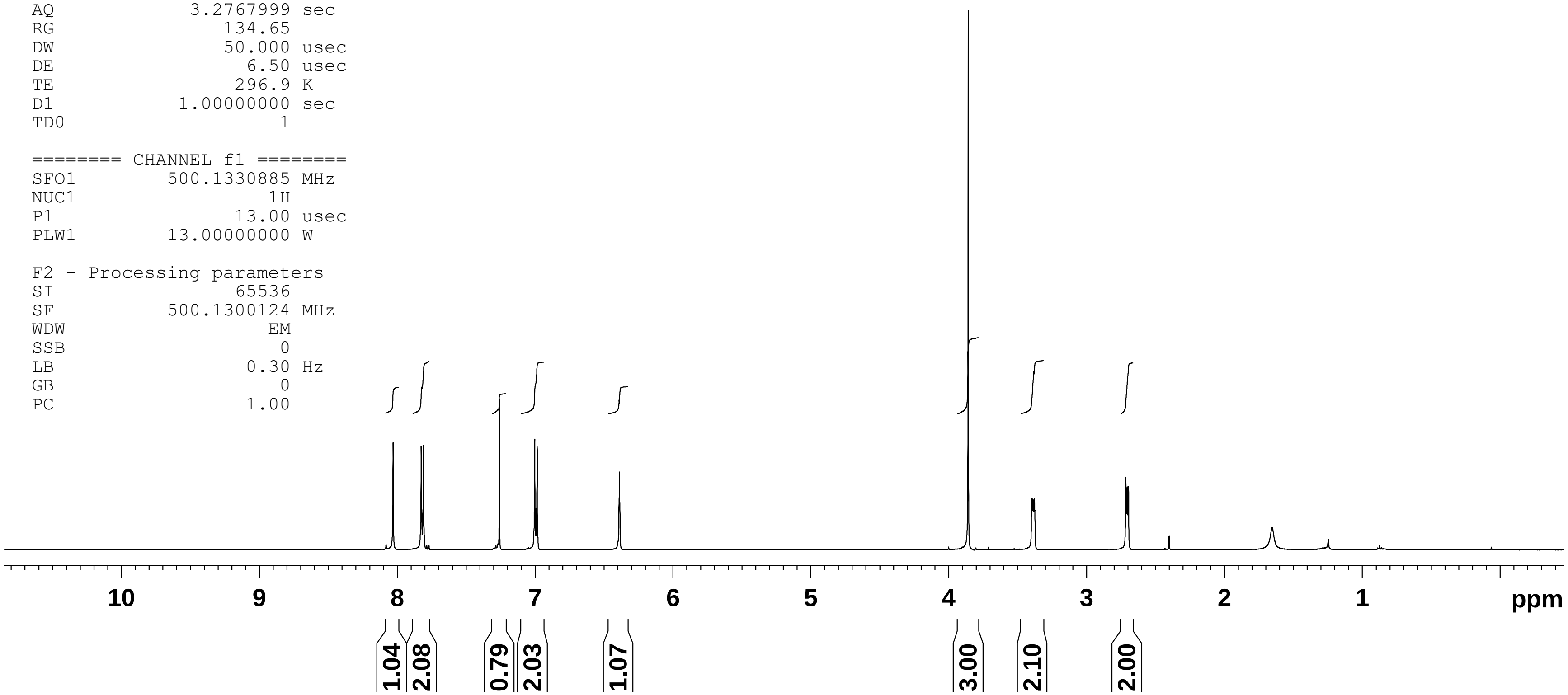

Figure S09: ^{1}H NMR spectrum of 3g.

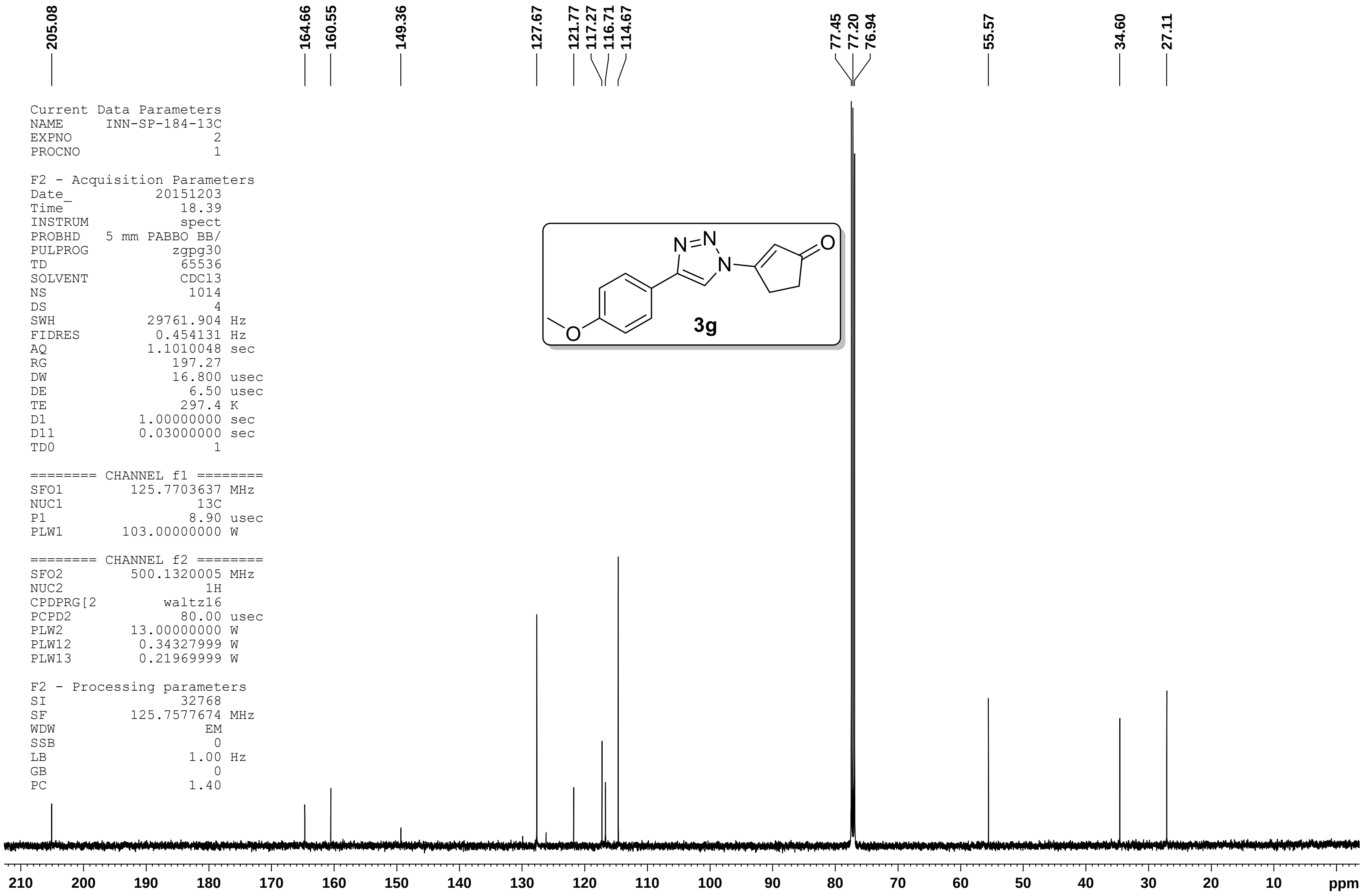

Figure S10: ^{13}C NMR spectrum of 3g.

```
Current Data Parameters
NAME     INN-SP-III-METHOXY-TRIAYALXNE-1H
EXPNO                 1
PROCNO                1

F2 - Acquisition Parameters
Date_          20180925
Time              19.36
INSTRUM           spect
PROBHD   5 mm PABBO BB/
PULPROG            zg30
TD                65536
SOLVENT           CDCl3
NS                   10
DS                    0
SWH           10000.000 Hz
FIDRES         0.152588 Hz
AQ            3.2767999 sec
RG               119.07
DW               50.000 usec
DE                 6.50 usec
TE                297.8 K
D1           1.00000000 sec
TD0                   1

======== CHANNEL f1 ========
SFO1        500.1330885 MHz
NUC1                 1H
P1                13.35 usec
PLW1        16.00000000 W

F2 - Processing parameters
SI                65536
SF          500.1300133 MHz
WDW                  EM
SSB                   0
LB                 0.30 Hz
GB                    0
PC                 1.00
```

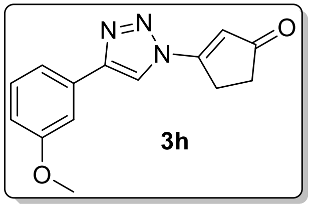

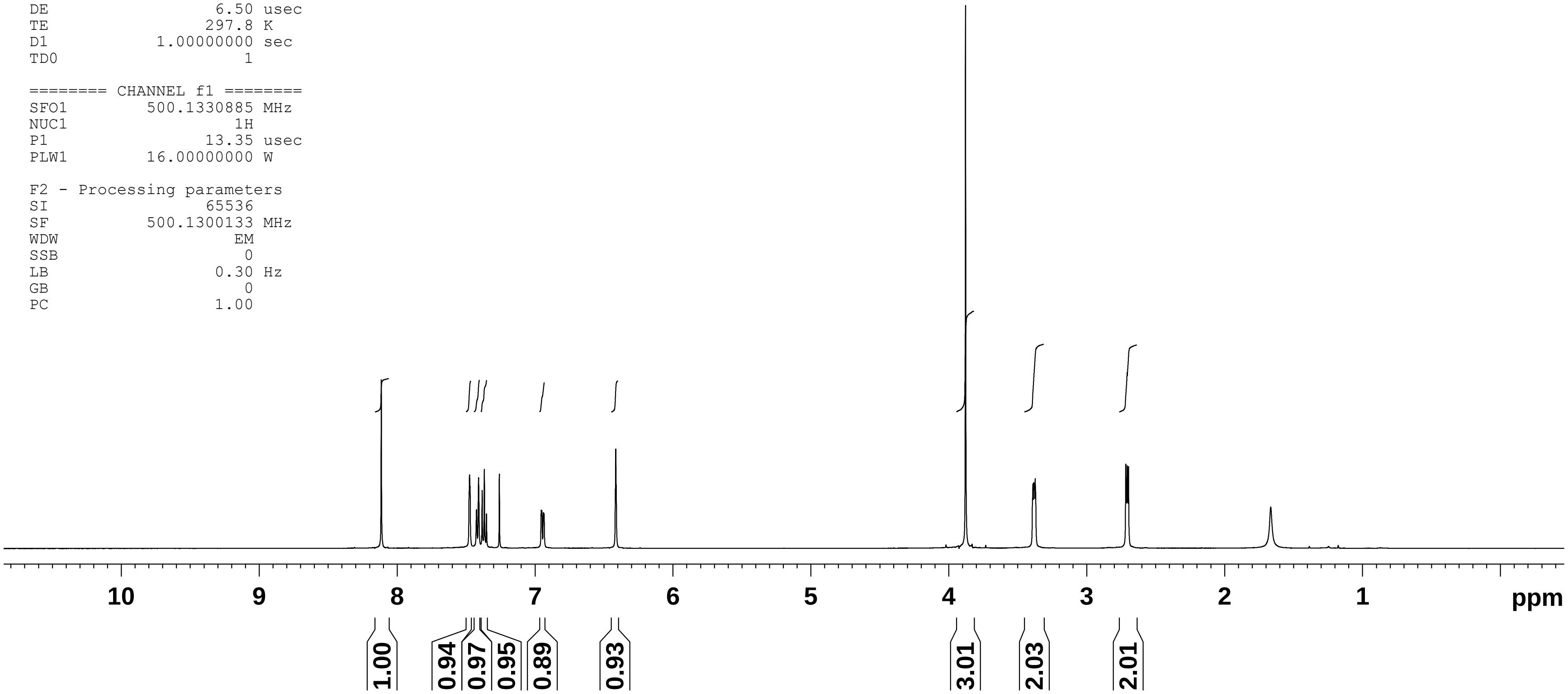

Figure S11: ^{1}H NMR spectrum of 3h.

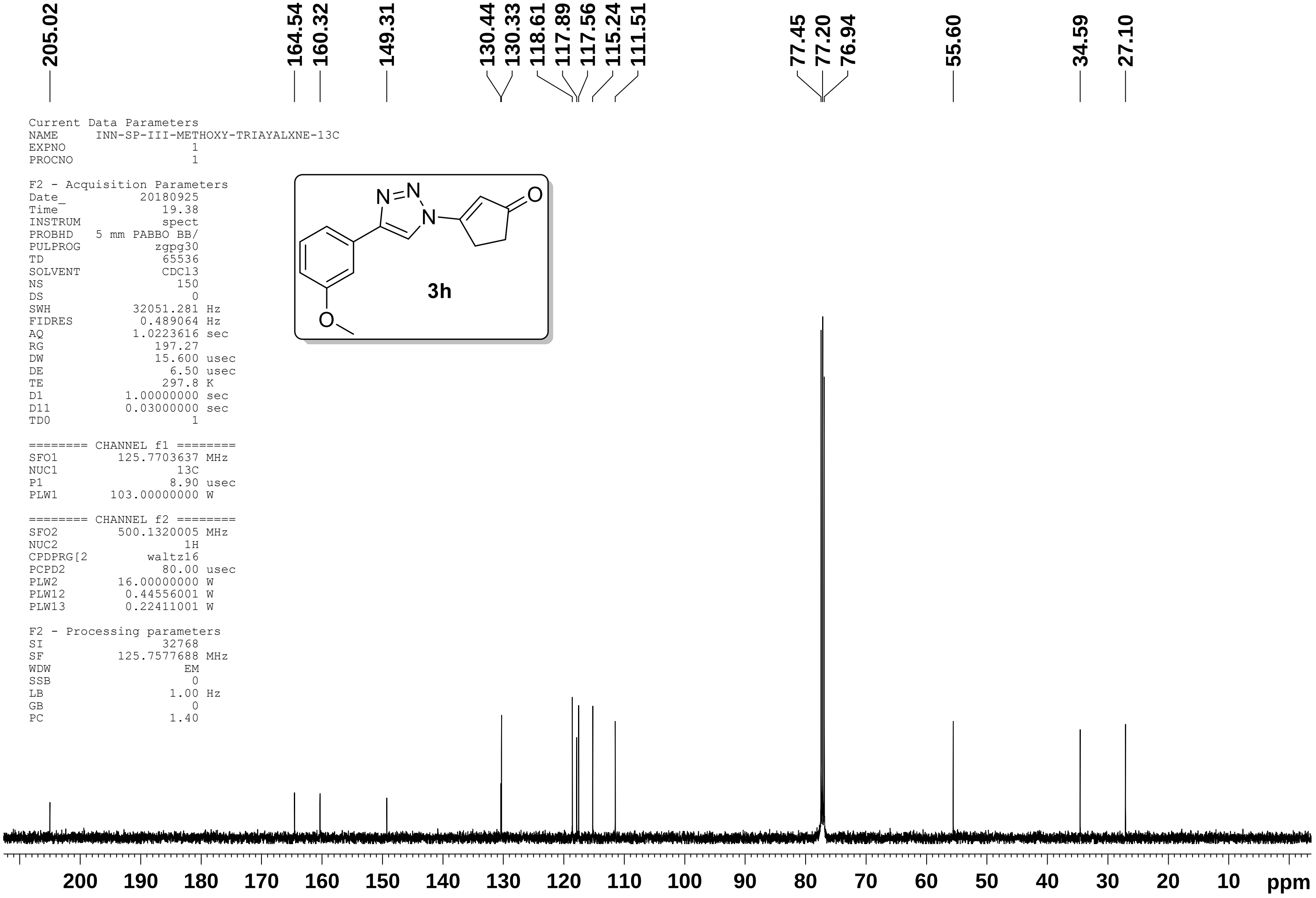

Figure S12: ^{13}C NMR spectrum of 3h.

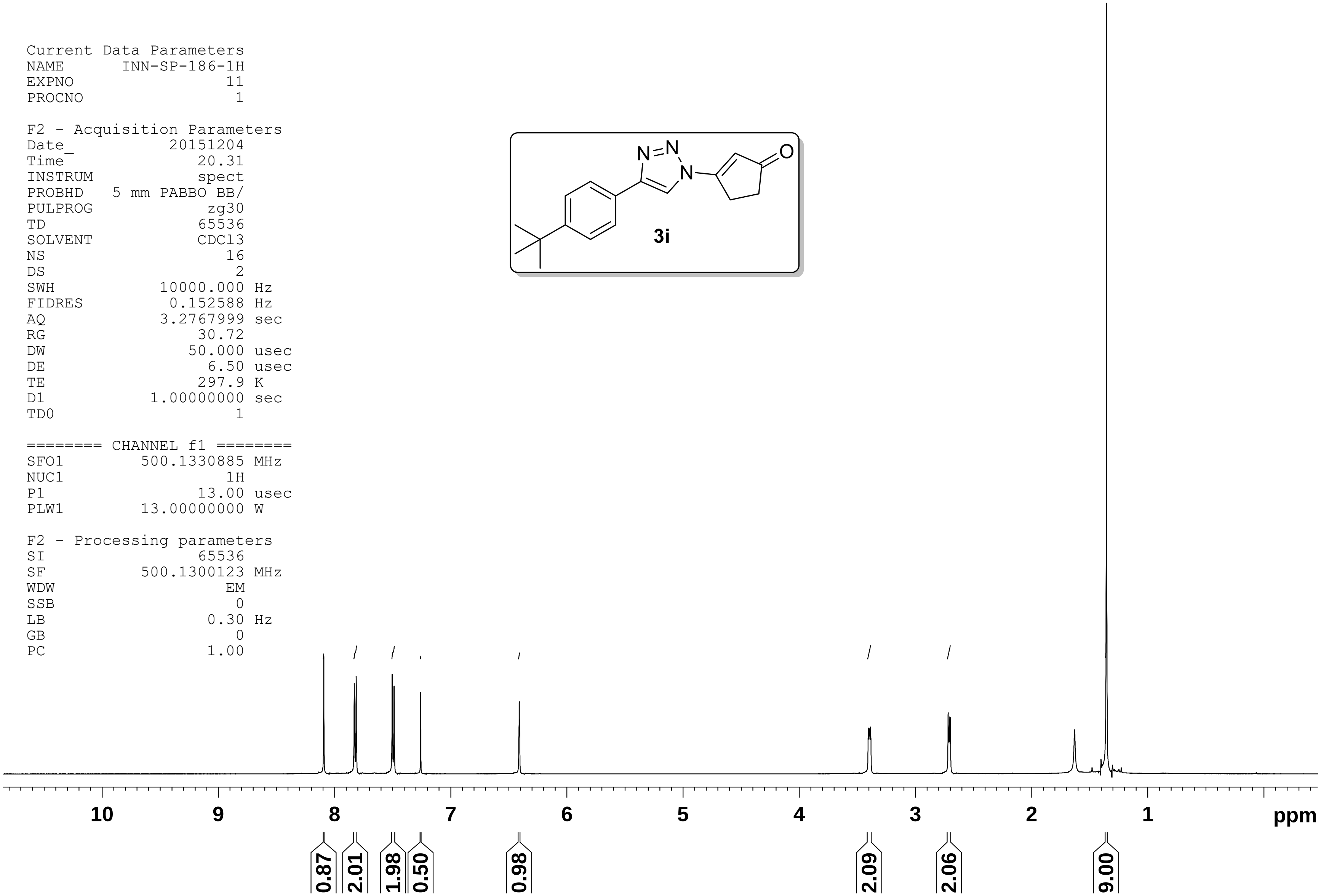

Figure S13: ^{1}H NMR spectrum of 3i.

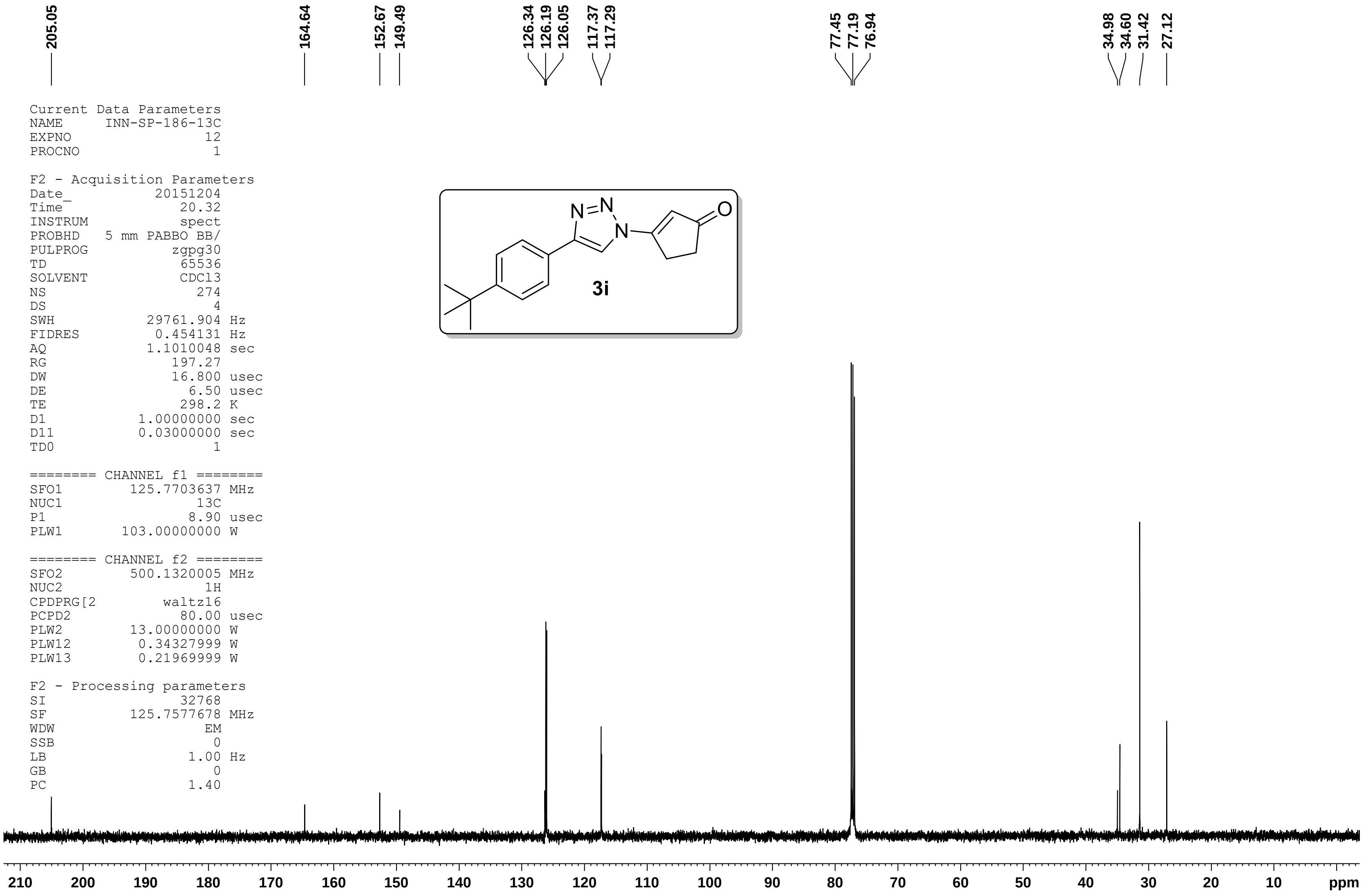

Figure S14: ^{13}C NMR spectrum of 3i.

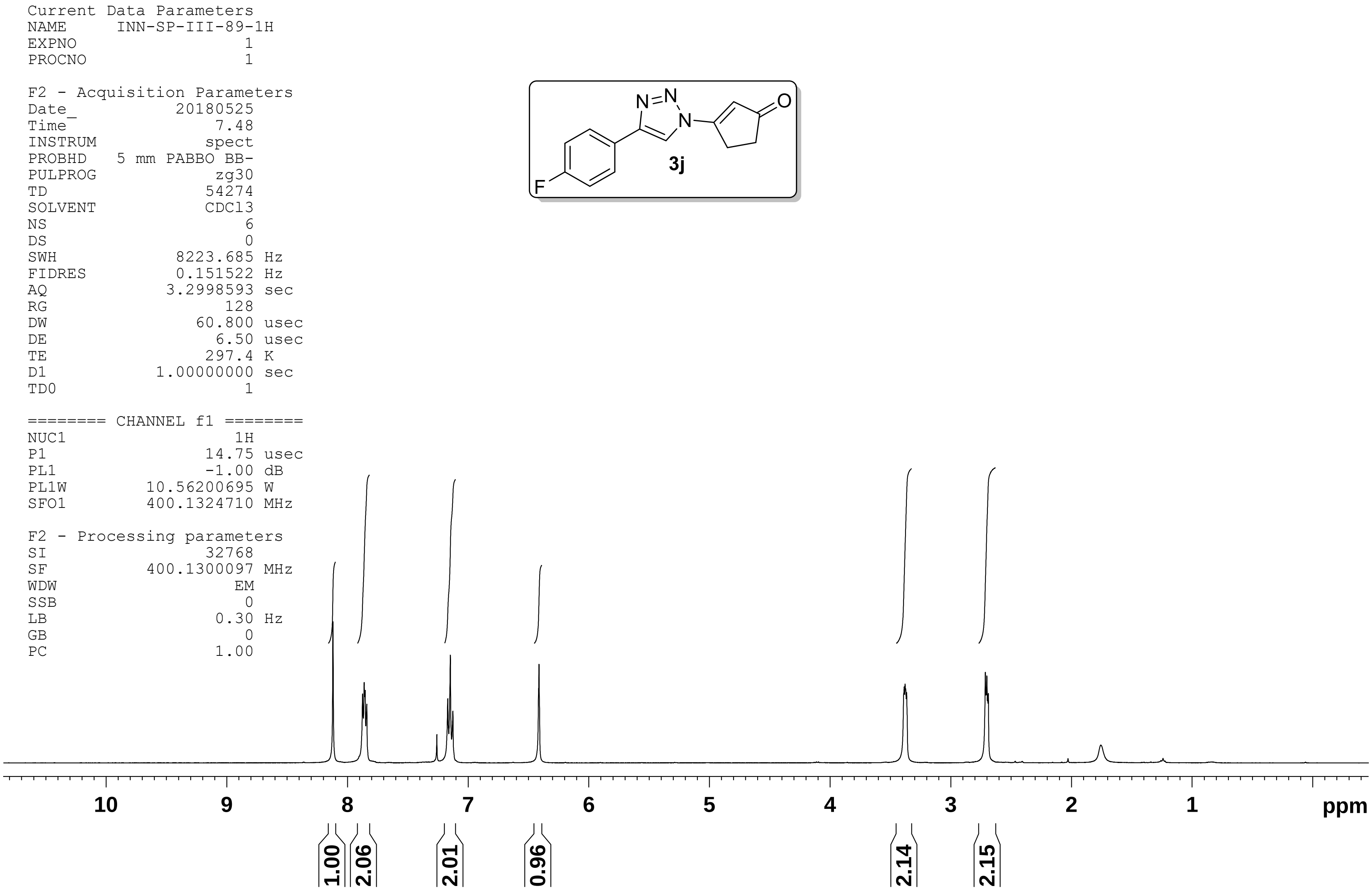

Figure S15: ^{1}H NMR spectrum of 3j.

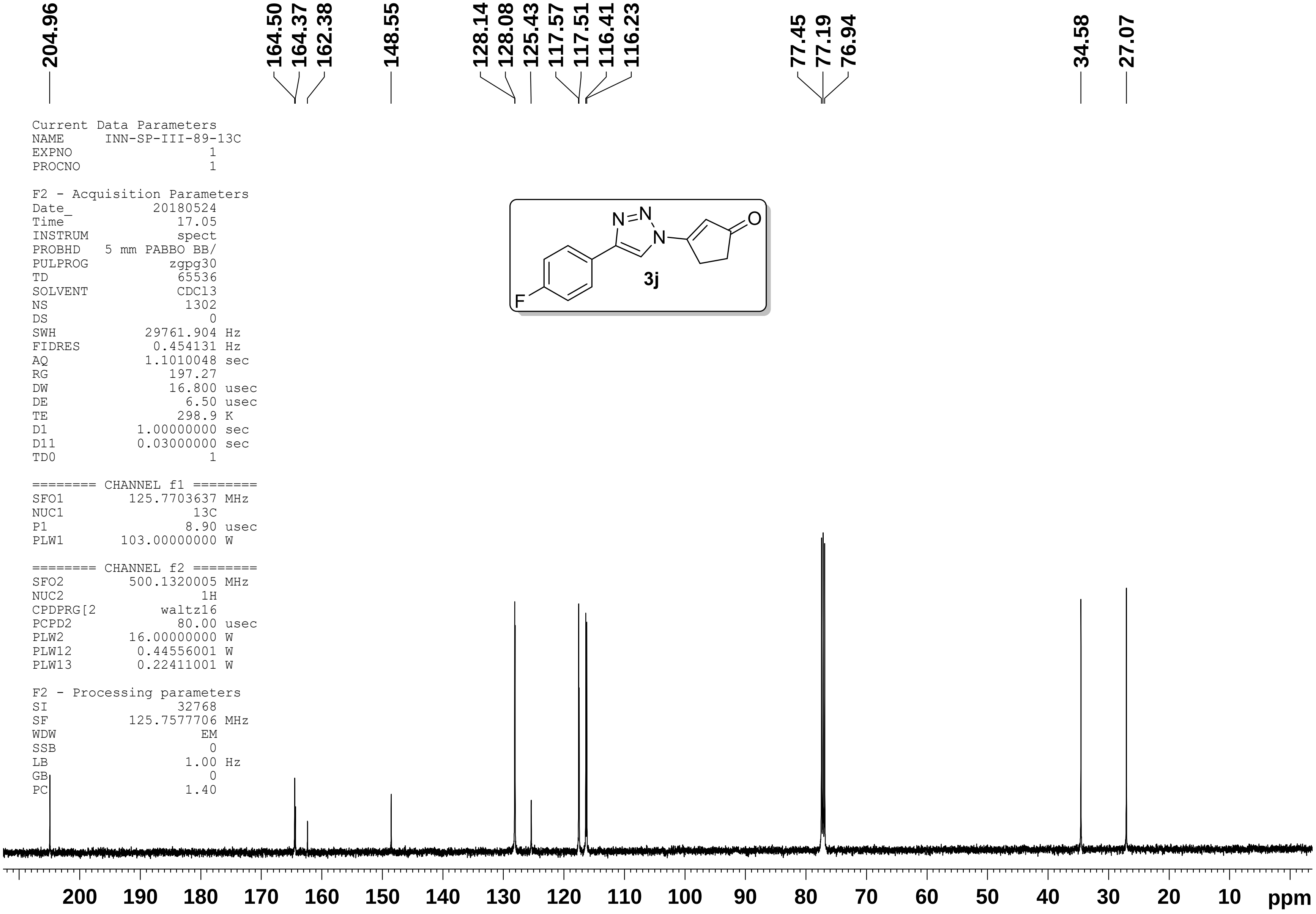

Figure S16: ^{13}C NMR spectrum of 3j.

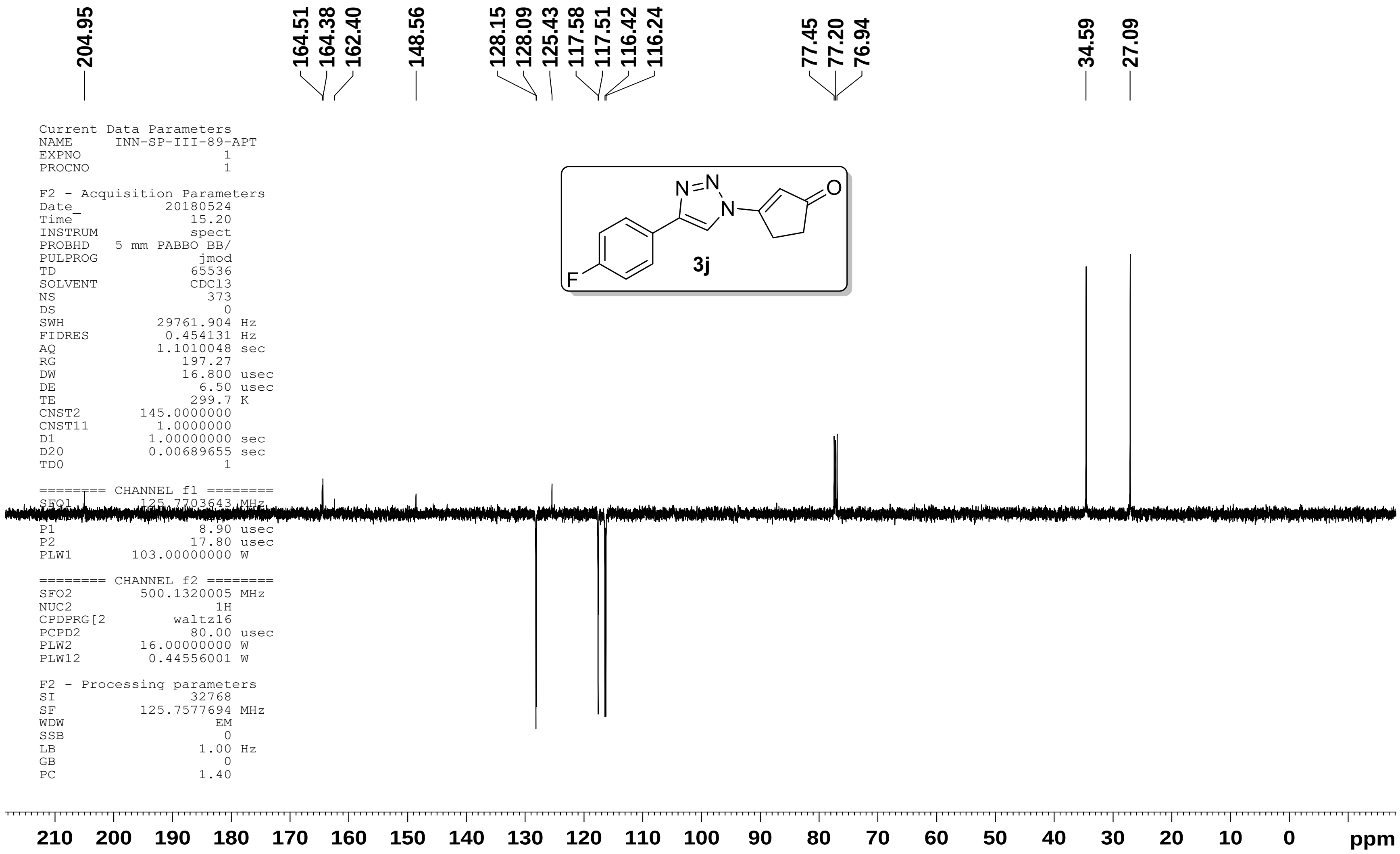

Figure S17: ^{13}C-APT NMR spectrum of 3j.

```
Current Data Parameters
NAME      INN-SP-III-89-19F
EXPNO                     1
PROCNO                    1

F2 - Acquisition Parameters
Date_          20180524
Time              15.37
INSTRUM           spect
PROBHD   5 mm PABBO BB/
PULPROG          zgflqn
TD               131072
SOLVENT           CDCl3
NS                   13
DS                    0
SWH          113636.367 Hz
FIDRES         0.866977 Hz
AQ            0.5767168 sec
RG               197.27
DW                4.400 usec
DE                 6.50 usec
TE                299.1 K
D1           1.00000000 sec
TD0                   1

======== CHANNEL f1 ========
SFO1        470.5453180 MHz
NUC1                19F
P1                19.75 usec
PLW1        55.00000000 W

F2 - Processing parameters
SI                65536
SF          470.5923770 MHz
WDW                  EM
SSB                   0
LB                 0.30 Hz
GB                    0
PC                 1.00
```

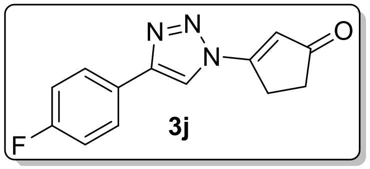

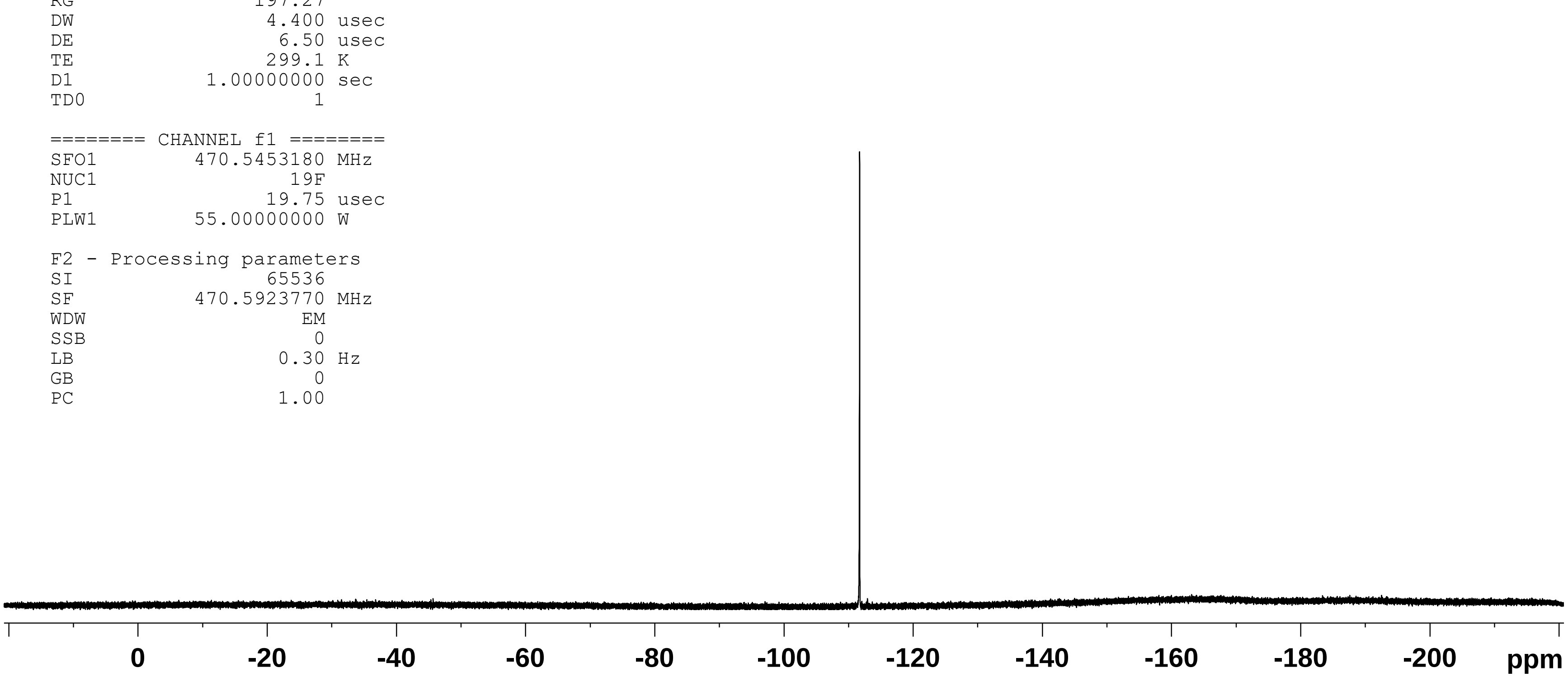

Figure S18: ^{19}F NMR spectrum of 3j.

```
Current Data Parameters
NAME      INN-SP-III-85-1H
EXPNO                    1
PROCNO                   1

F2 - Acquisition Parameters
Date_           20180524
Time               13.45
INSTRUM            spect
PROBHD   5 mm PABBO BB/
PULPROG             zg30
TD                 65536
SOLVENT            CDCl3
NS                     9
DS                     0
SWH            10000.000 Hz
FIDRES          0.152588 Hz
AQ             3.2767999 sec
RG                177.33
DW                50.000 usec
DE                  6.50 usec
TE                 300.1 K
D1            1.00000000 sec
TD0                    1

======== CHANNEL f1 ========
SFO1         500.1330885 MHz
NUC1                  1H
P1                 13.35 usec
PLW1         16.00000000 W

F2 - Processing parameters
SI                 65536
SF           500.1300132 MHz
WDW                   EM
SSB                    0
LB                  0.30 Hz
GB                     0
PC                  1.00
```

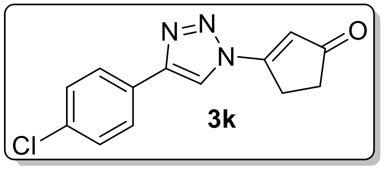

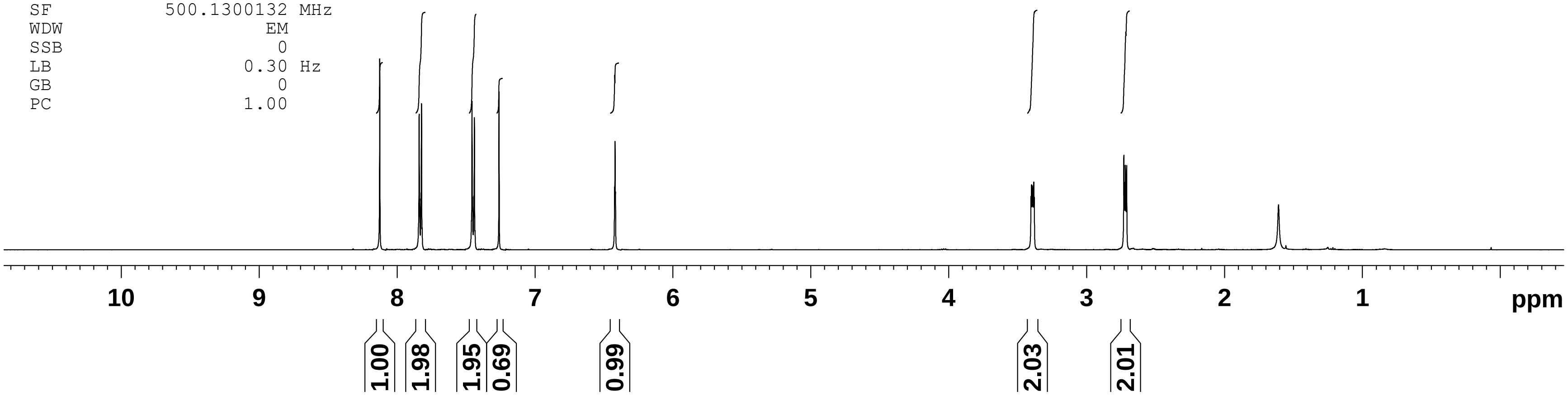

Figure S19: ^{1}H NMR spectrum of 3k.

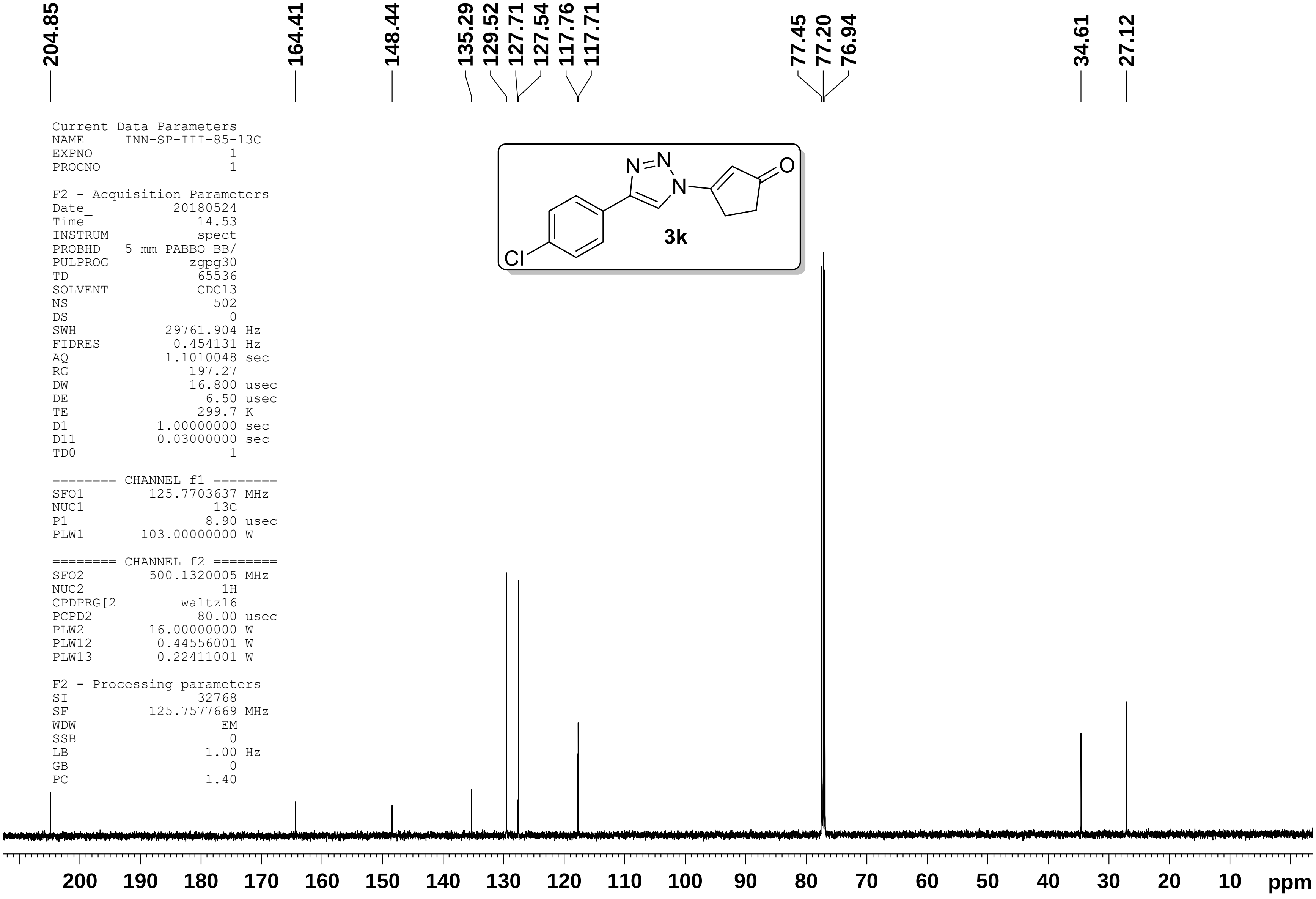

Figure S20: ^{13}C NMR spectrum of 3k.

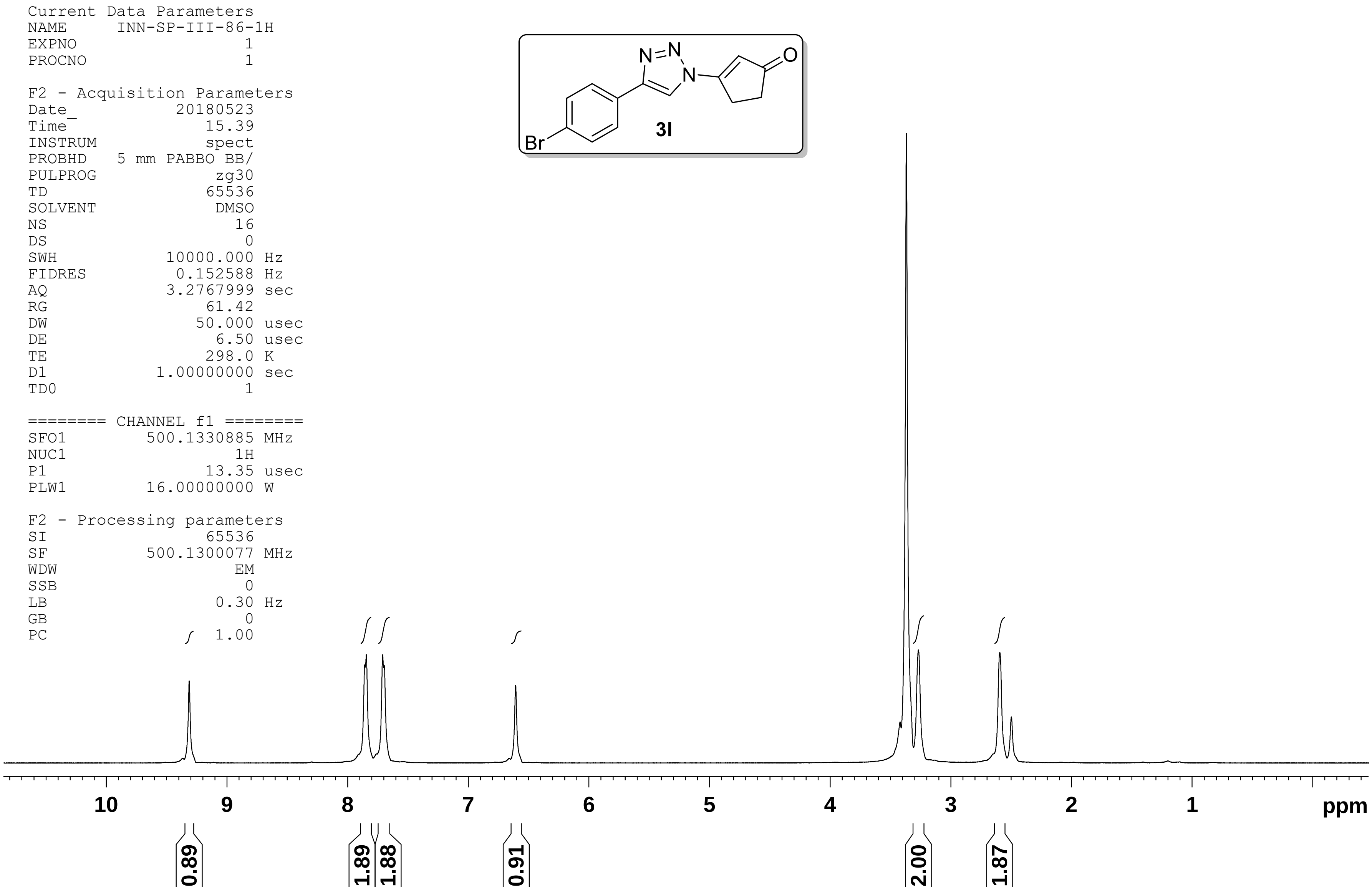

Figure S21: ^{1}H NMR spectrum of 3l.

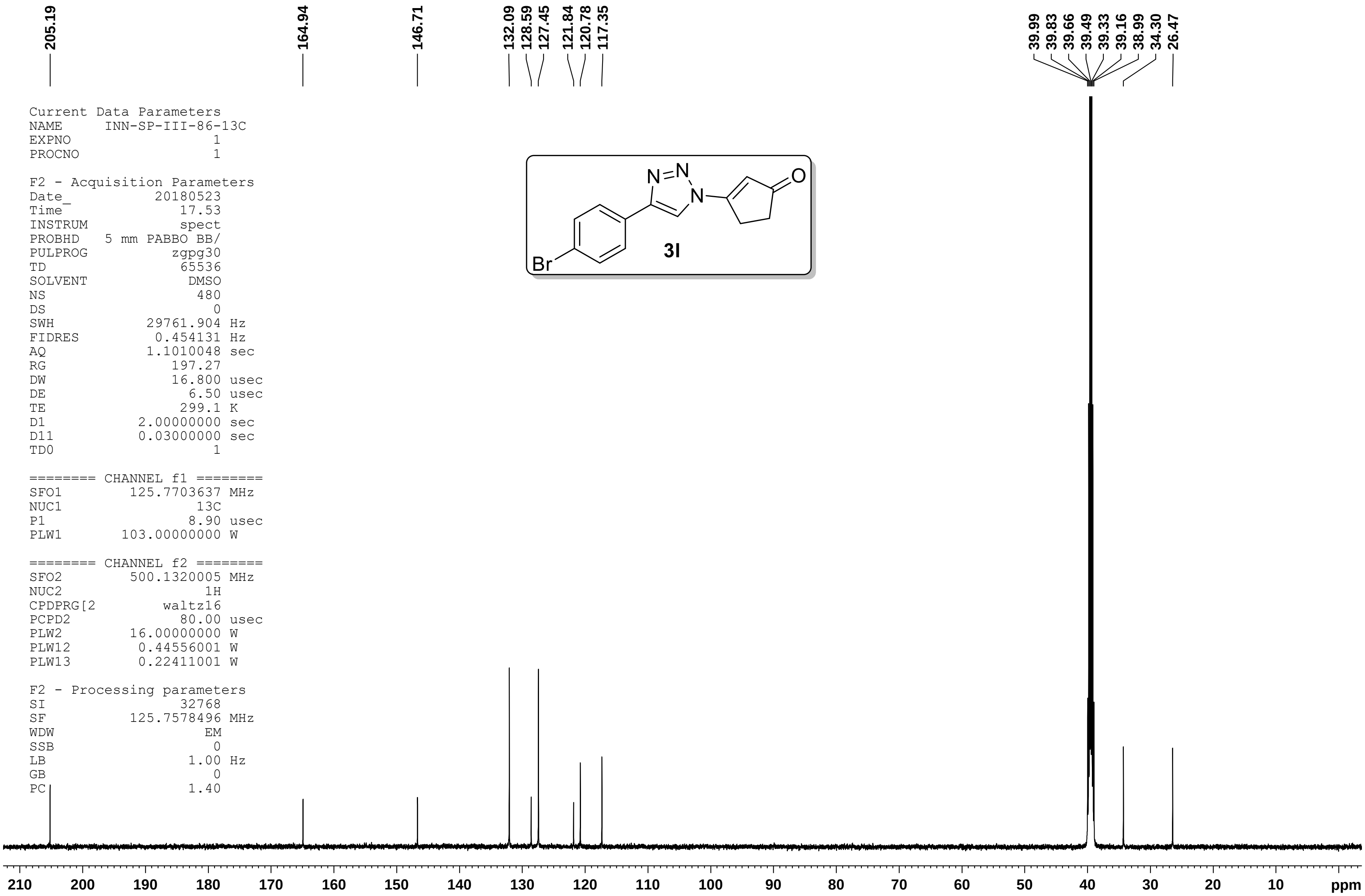

Figure S22: ^{13}C NMR spectrum of 3l.

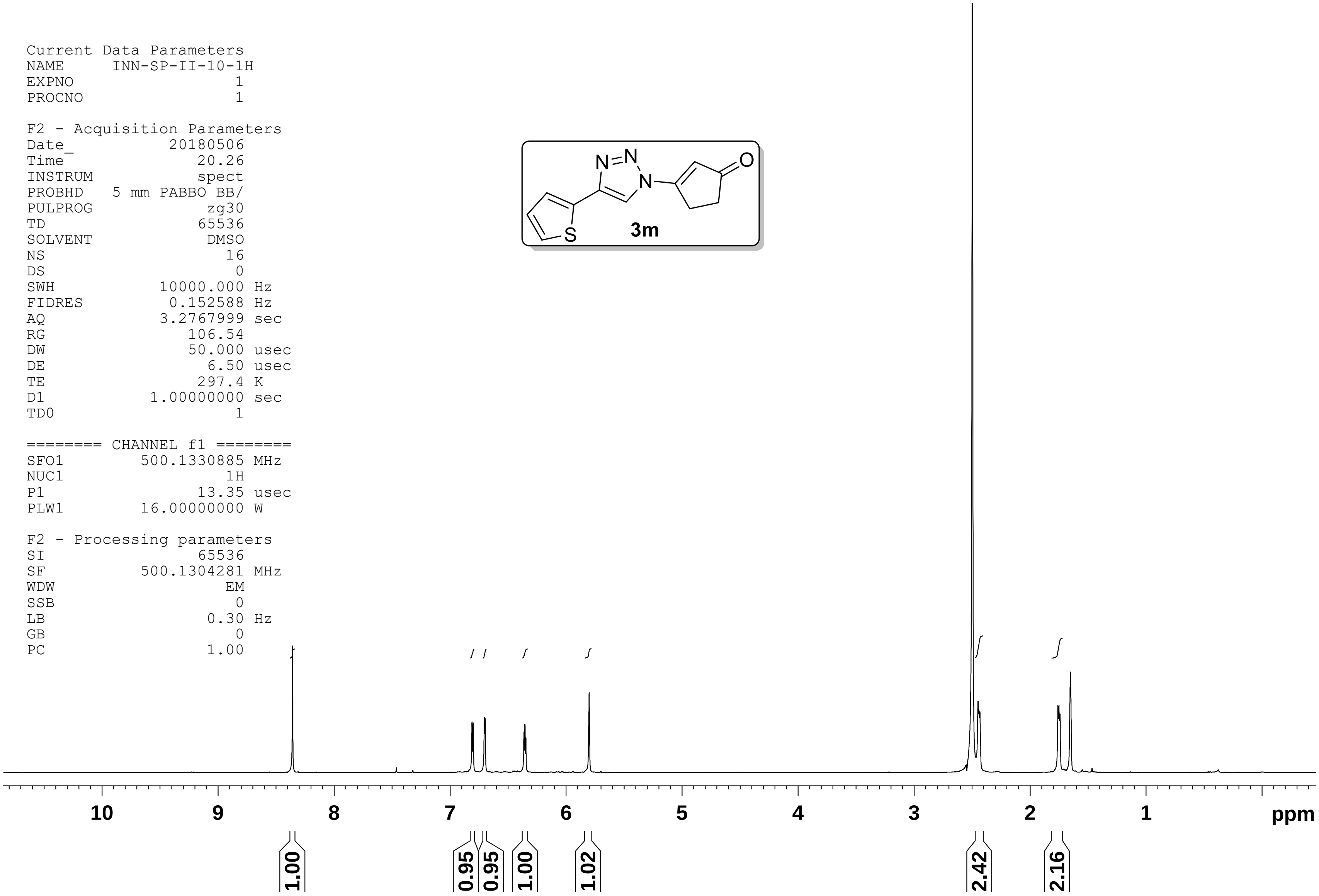

Figure S23: ^{1}H NMR spectrum of 3m.

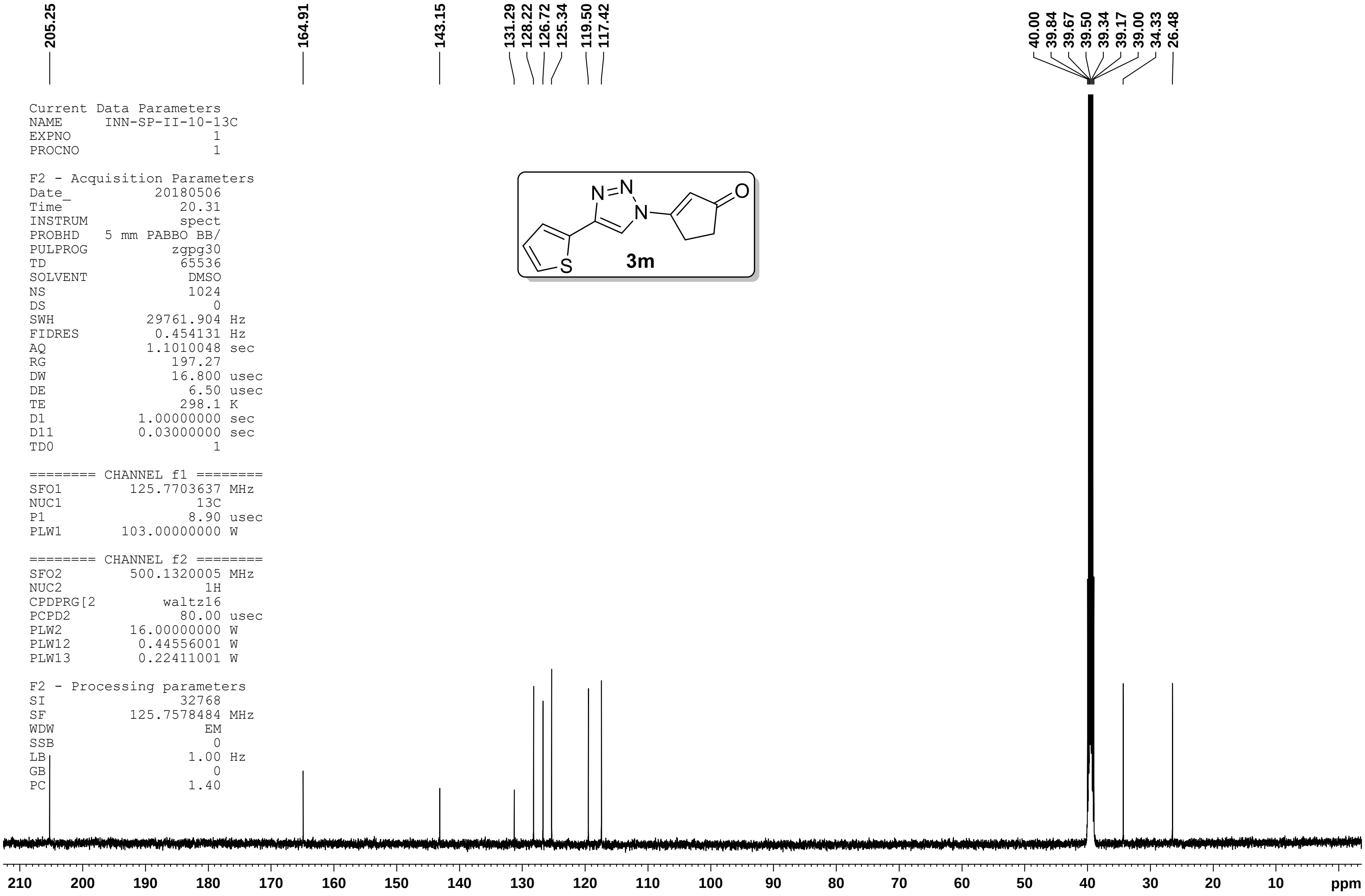

Figure S24: ^{13}C NMR spectrum of 3m.

```
Current Data Parameters
NAME      INN-SP-II-104-1H
EXPNO                    4
PROCNO                   1

F2 - Acquisition Parameters
Date_           20180417
Time               15.41
INSTRUM            spect
PROBHD   5 mm PABBO BB/
PULPROG             zg30
TD                 65536
SOLVENT            CDCl3
NS                    16
DS                     0
SWH            10000.000 Hz
FIDRES          0.152588 Hz
AQ             3.2767999 sec
RG                134.65
DW                50.000 usec
DE                  6.50 usec
TE                 298.2 K
D1            1.00000000 sec
TD0                    1

======== CHANNEL f1 ========
SFO1         500.1330885 MHz
NUC1                  1H
P1                 13.35 usec
PLW1         16.00000000 W

F2 - Processing parameters
SI                 65536
SF           500.1300131 MHz
WDW                   EM
SSB                    0
LB                  0.10 Hz
GB                     0
PC                  1.00
```

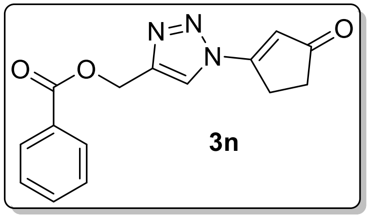

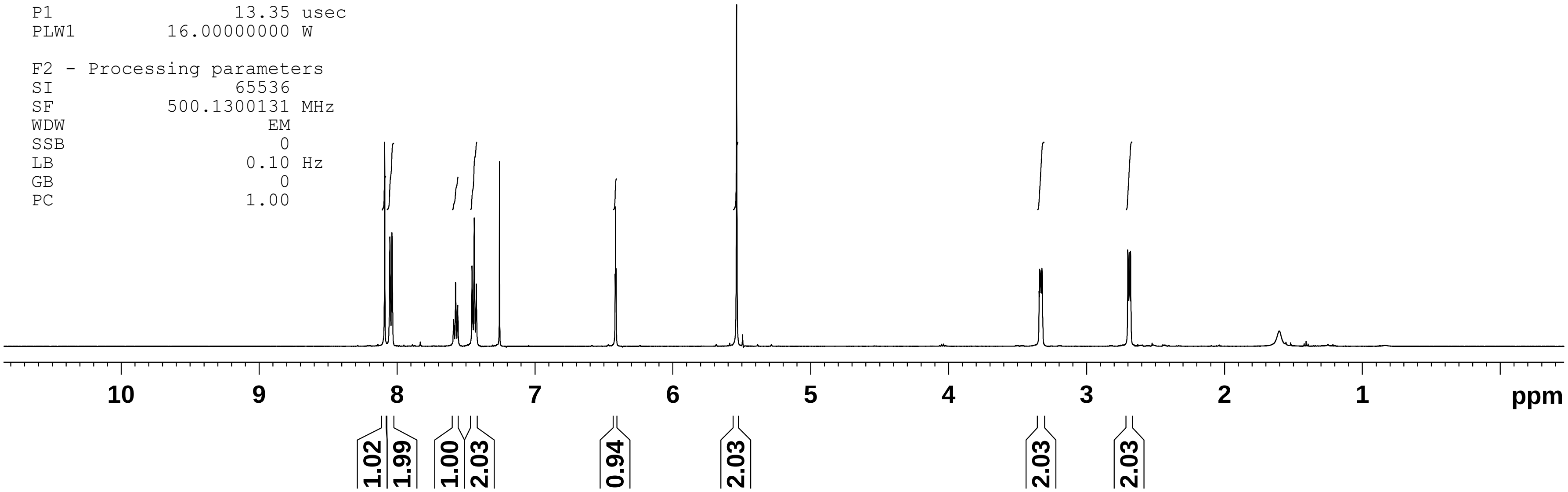

Figure S25: ^{1}H NMR spectrum of 3n.

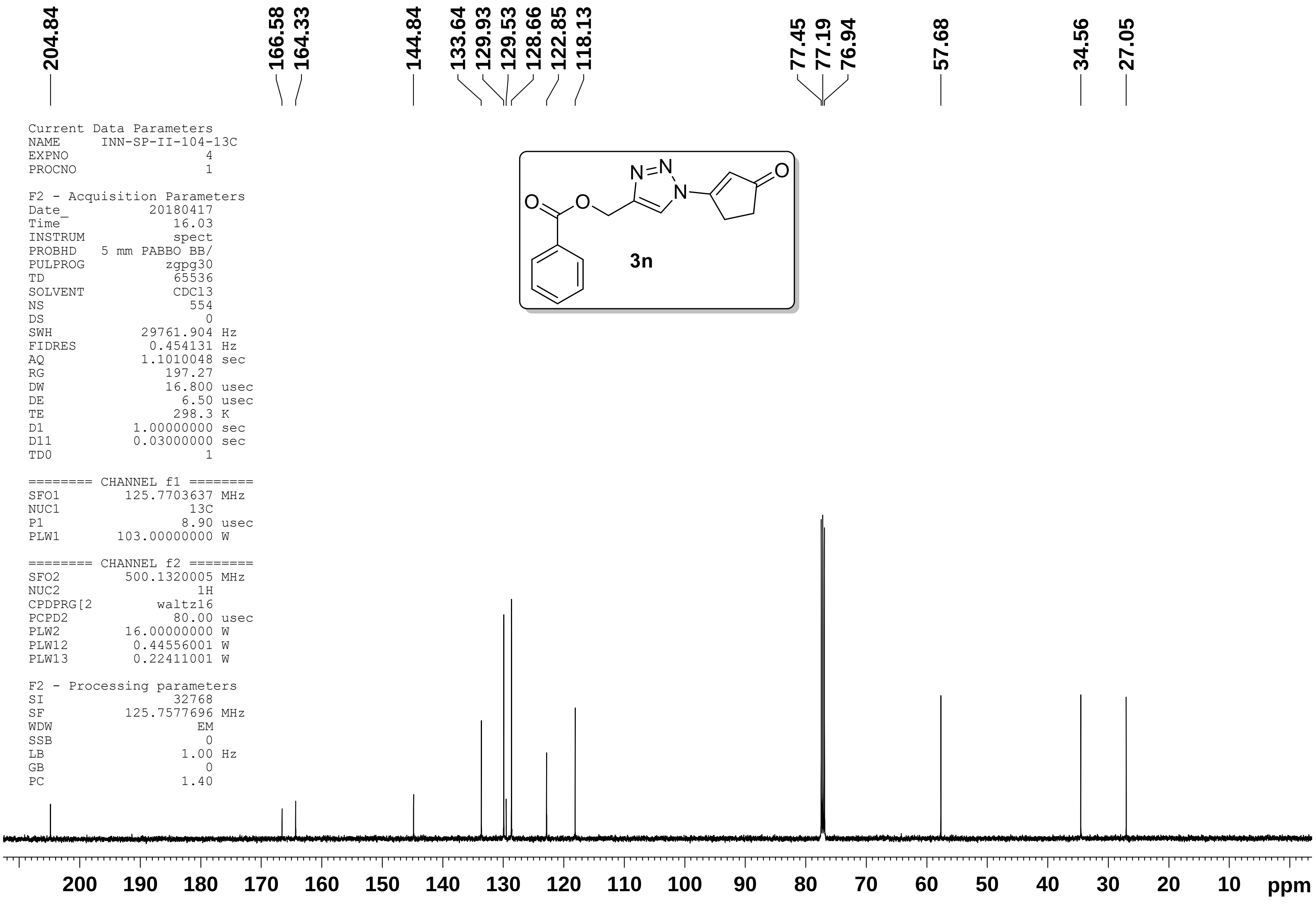

Figure S26: ^{13}C NMR spectrum of 3n.

```
Current Data Parameters
NAME     INN-SP-II-126-1H
EXPNO                   1
PROCNO                  1

F2 - Acquisition Parameters
Date_          20170210
Time              17.11
INSTRUM           spect
PROBHD   5 mm PABBO BB/
PULPROG            zg30
TD                65536
SOLVENT           CDCl3
NS                   10
DS                    2
SWH           10000.000 Hz
FIDRES         0.152588 Hz
AQ            3.2767999 sec
RG               106.54
DW               50.000 usec
DE                 6.50 usec
TE                297.9 K
D1           1.00000000 sec
TD0                   1

======== CHANNEL f1 ========
SFO1        500.1330885 MHz
NUC1                 1H
P1                13.00 usec
PLW1        13.00000000 W

F2 - Processing parameters
SI                65536
SF          500.1300131 MHz
WDW                  EM
SSB                   0
LB                 0.30 Hz
GB                    0
PC                 1.00
```

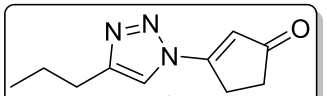

3o

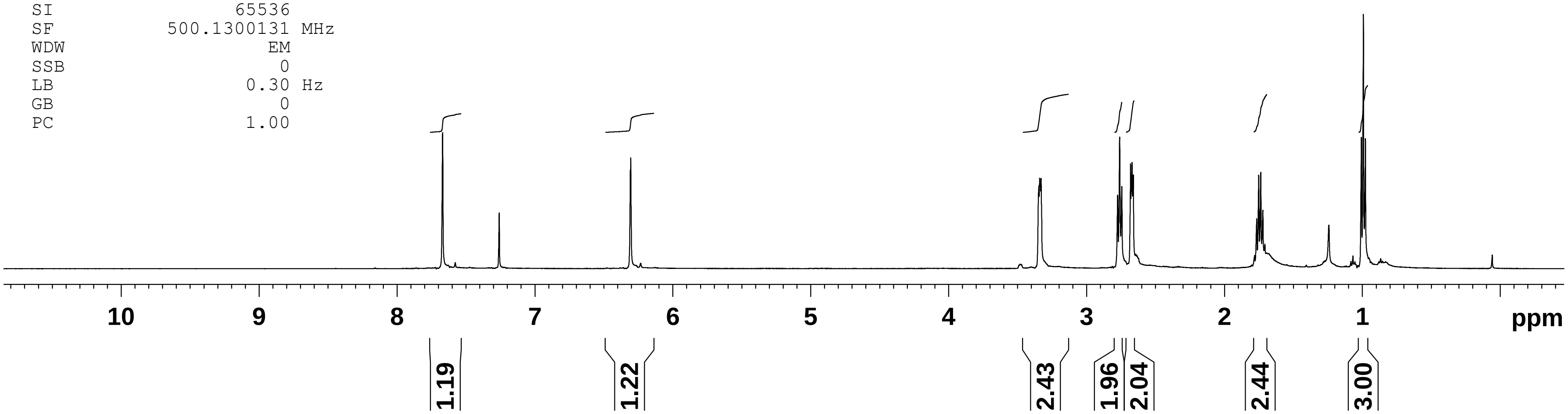

Figure S27: ^{1}H NMR spectrum of 3o.

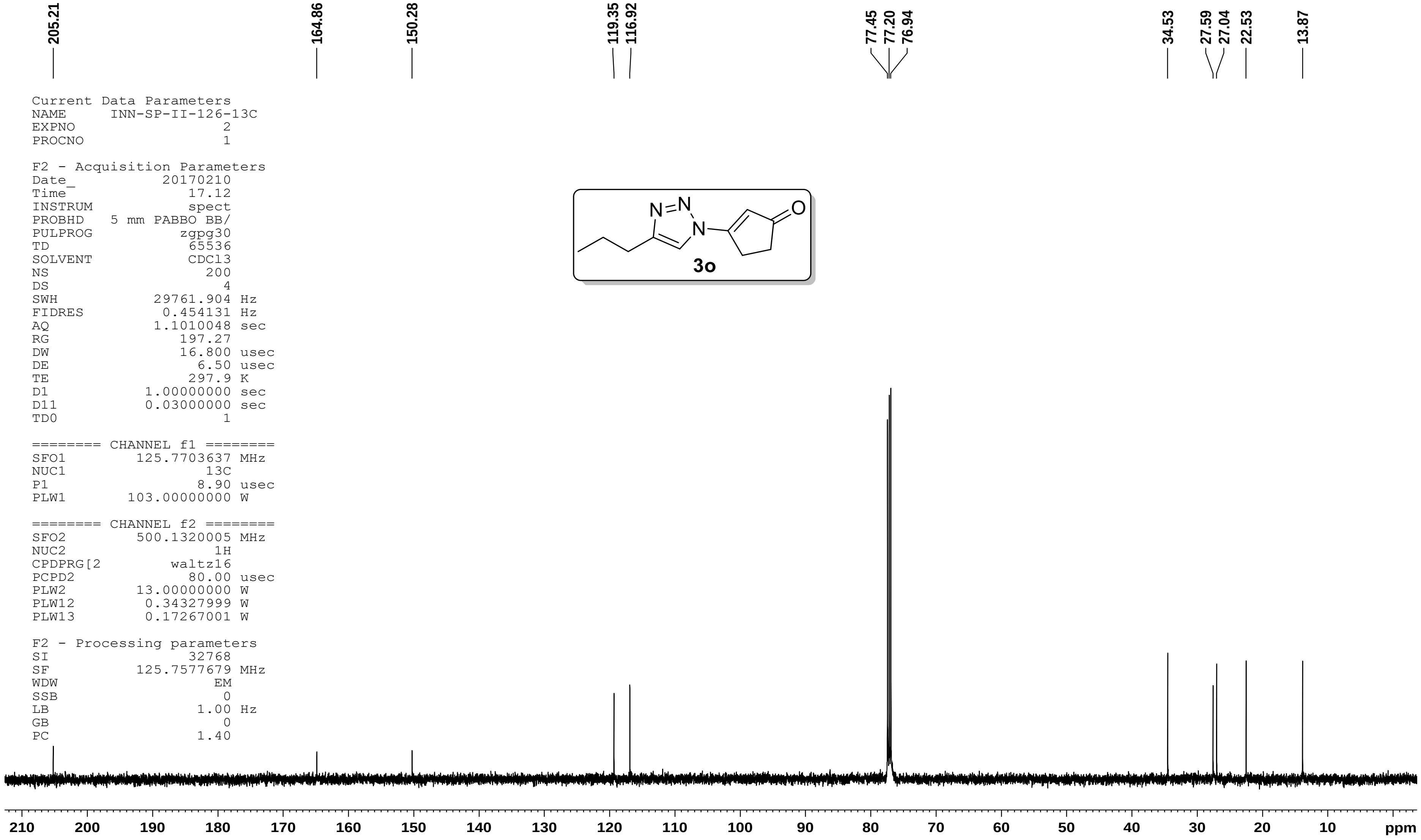

Figure S28: ^{13}C NMR spectrum of 3o.

```
Current Data Parameters
NAME     INN-SP-III-81-1H
EXPNO                   1
PROCNO                  1

F2 - Acquisition Parameters
Date_          20180605
Time               5.33
INSTRUM           spect
PROBHD   5 mm PABBO BB-
PULPROG            zg30
TD                54274
SOLVENT           CDCl3
NS                    7
DS                    0
SWH            8223.685 Hz
FIDRES         0.151522 Hz
AQ            3.2998593 sec
RG                  228
DW               60.800 usec
DE                 6.50 usec
TE                513.2 K
D1           1.00000000 sec
TD0                   1

======== CHANNEL f1 ========
NUC1                 1H
P1                14.75 usec
PL1               -1.00 dB
PL1W        10.56200695 W
SFO1        400.1324710 MHz

F2 - Processing parameters
SI                32768
SF          400.1300102 MHz
WDW                  EM
SSB                   0
LB                 0.30 Hz
GB                    0
PC                 1.00
```

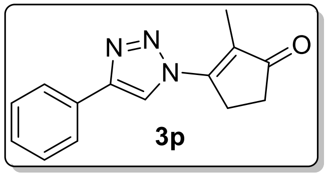

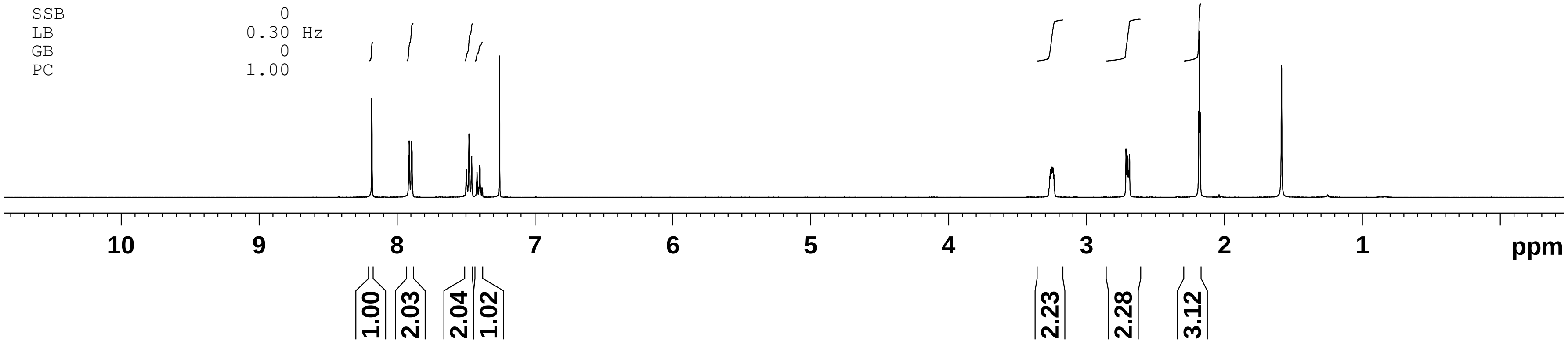

Figure S29: ^{1}H NMR spectrum of 3p.

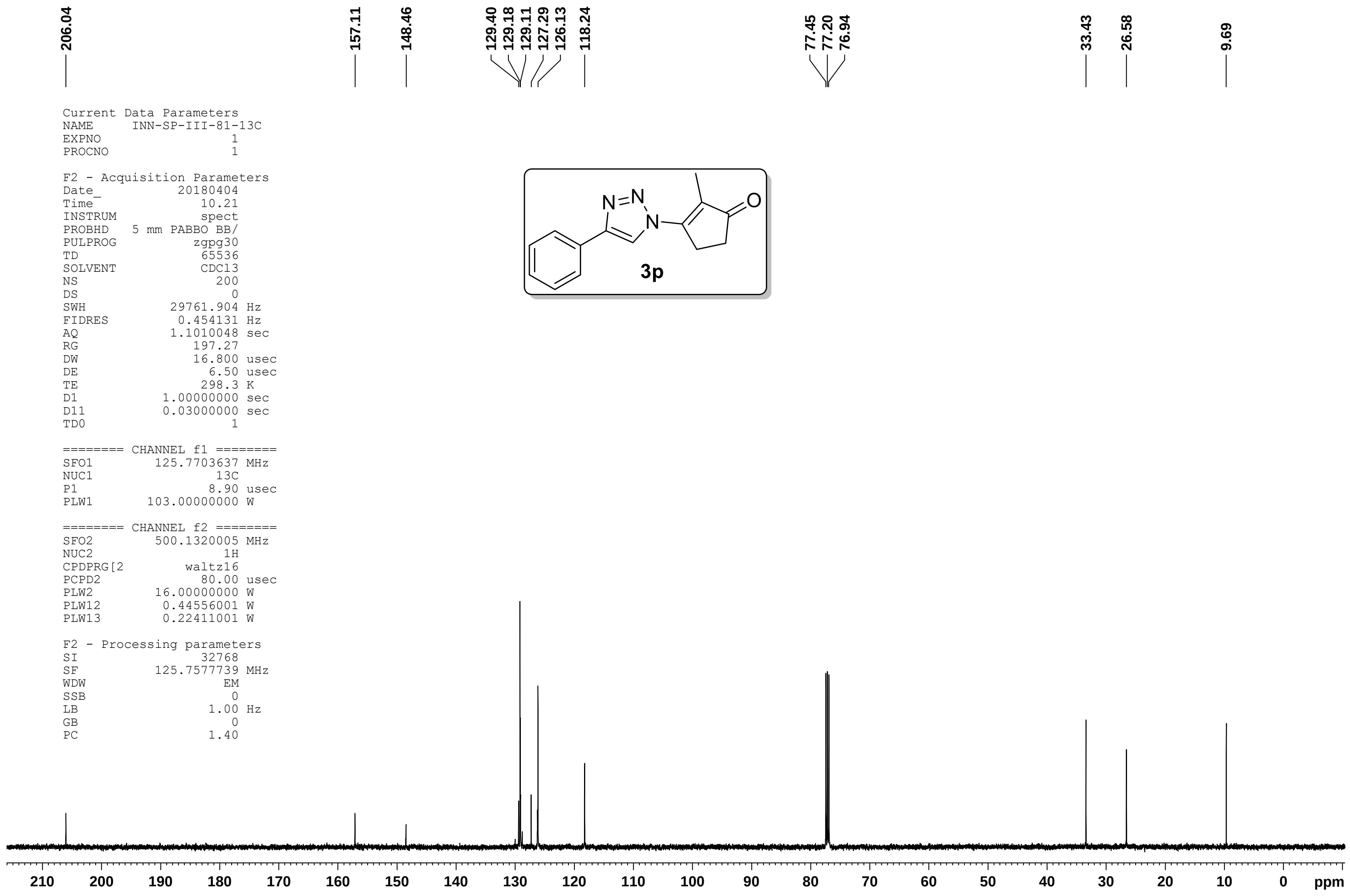

Figure S30: ^{13}C NMR spectrum of 3p.

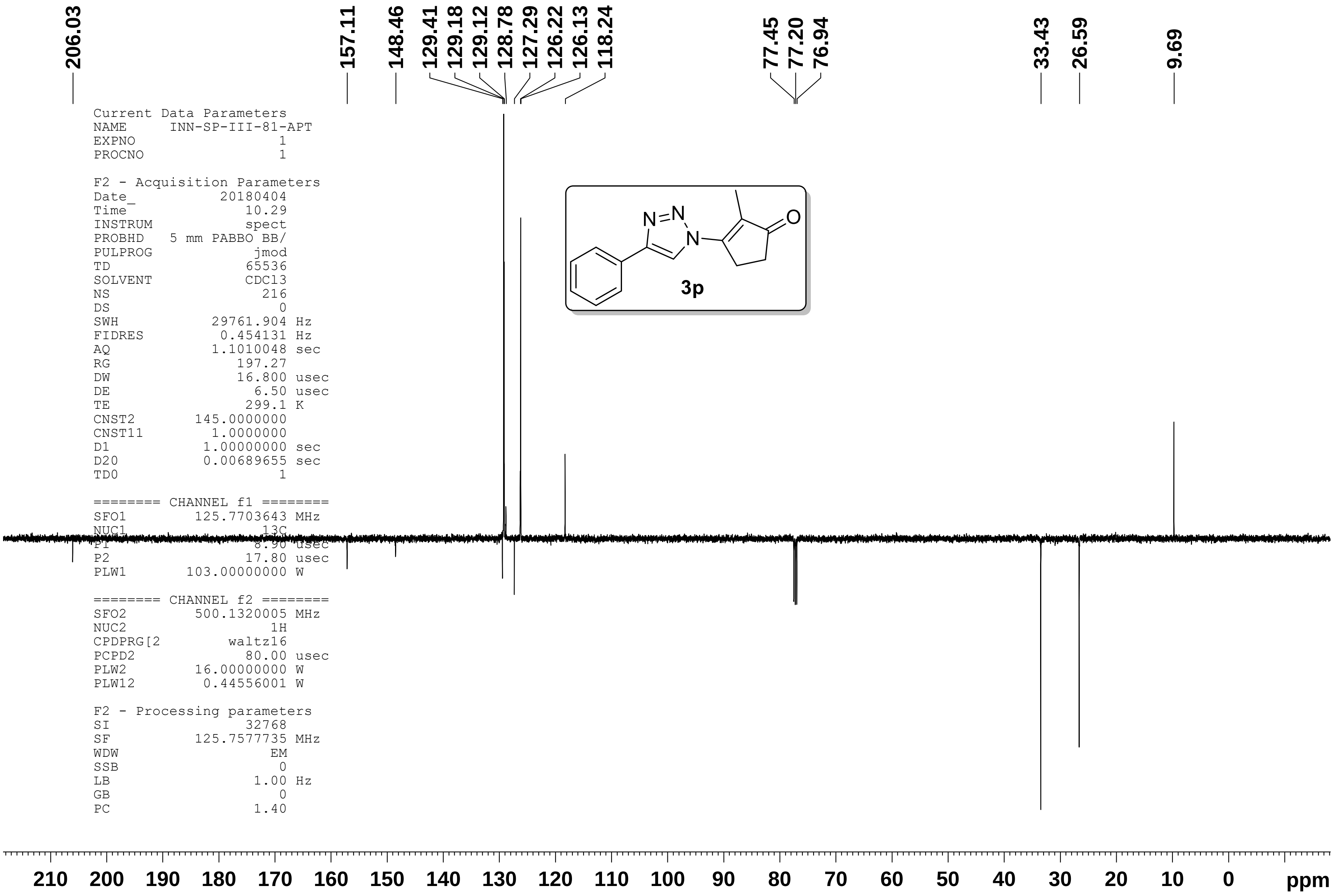

Figure S31: ^{13}C-APT NMR spectrum of 3p.

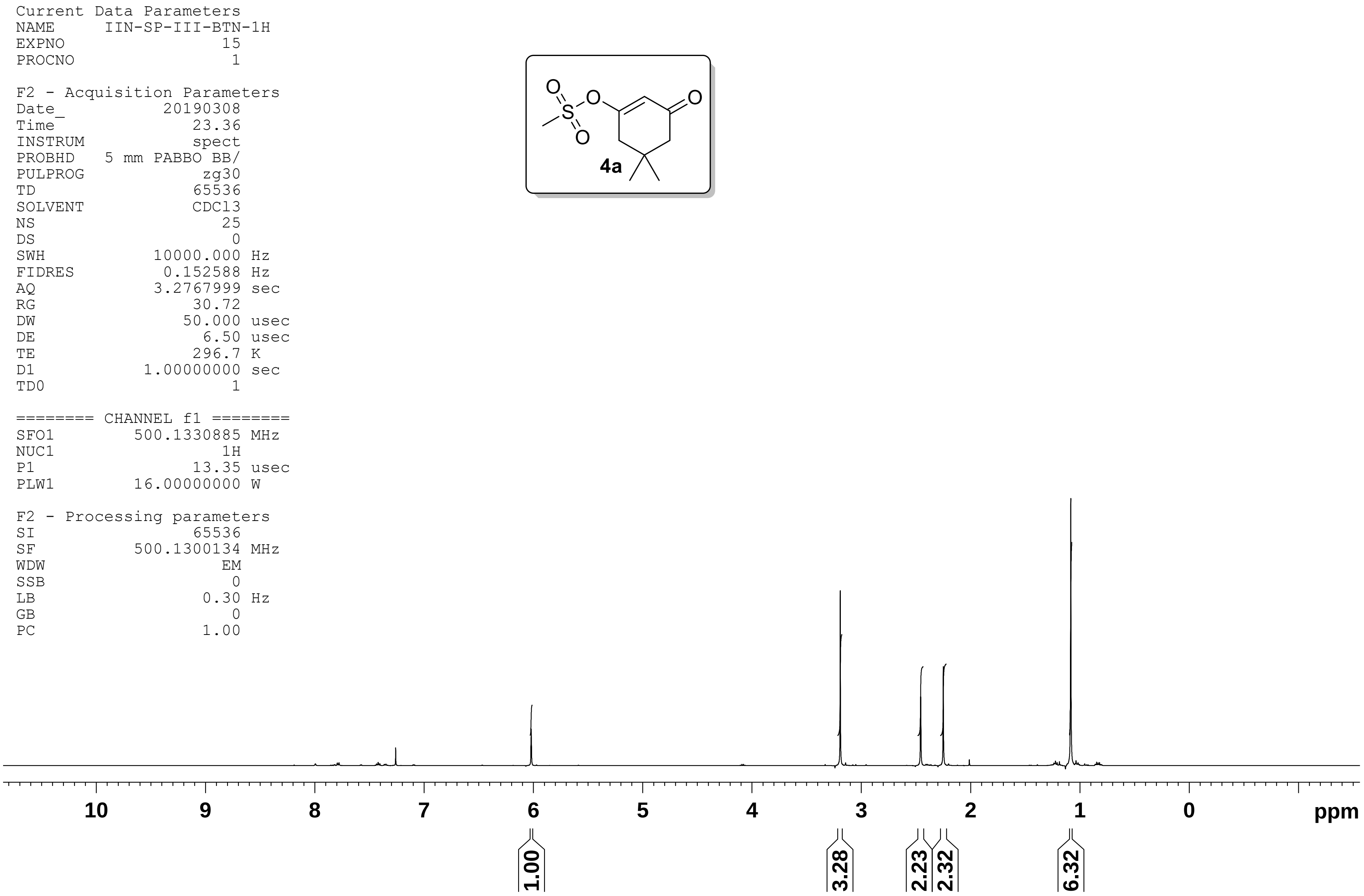

Figure S32: ^{1}H NMR spectrum of 4a.

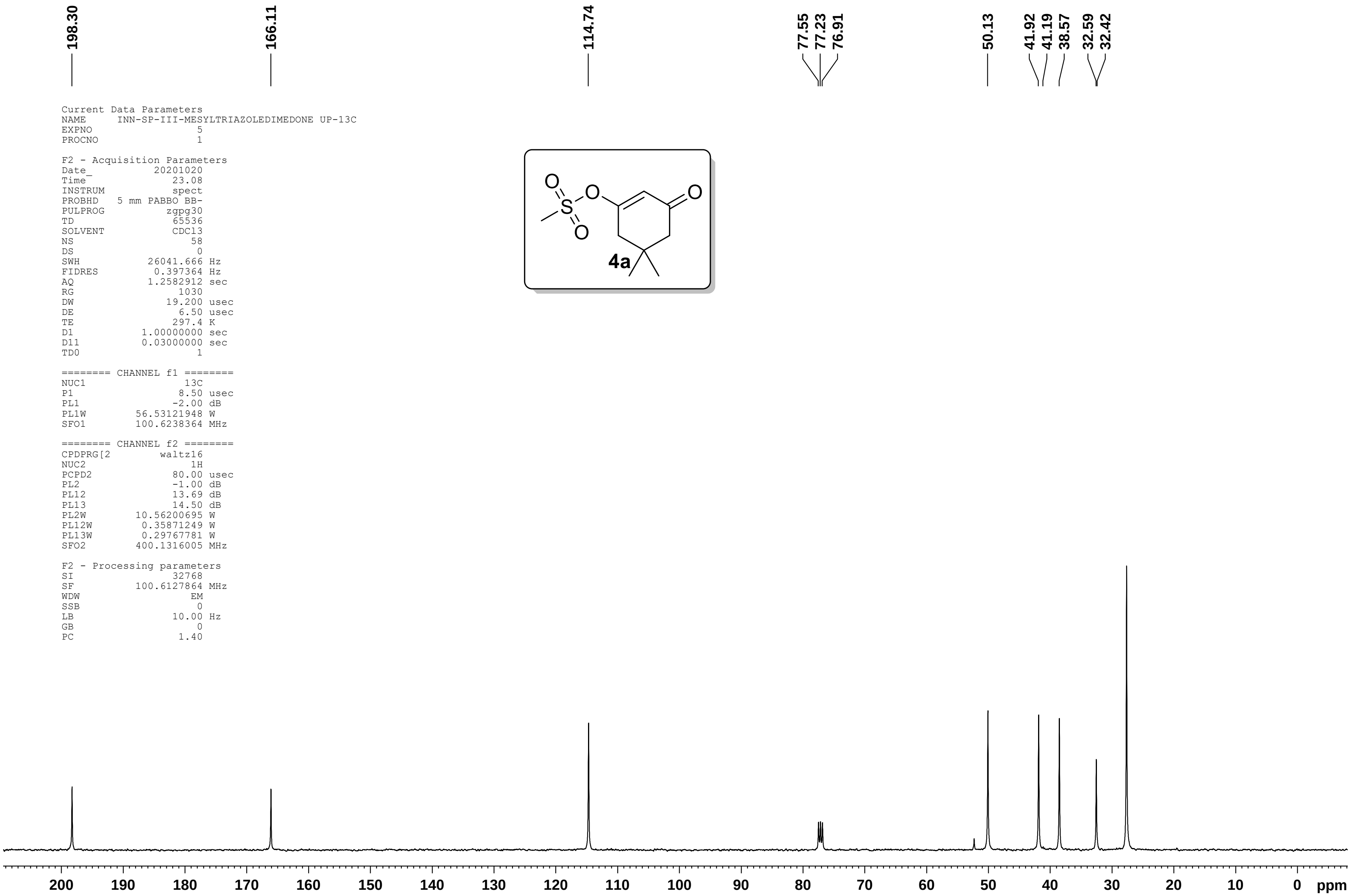

Figure S33: ^{13}C NMR spectrum of 4a.

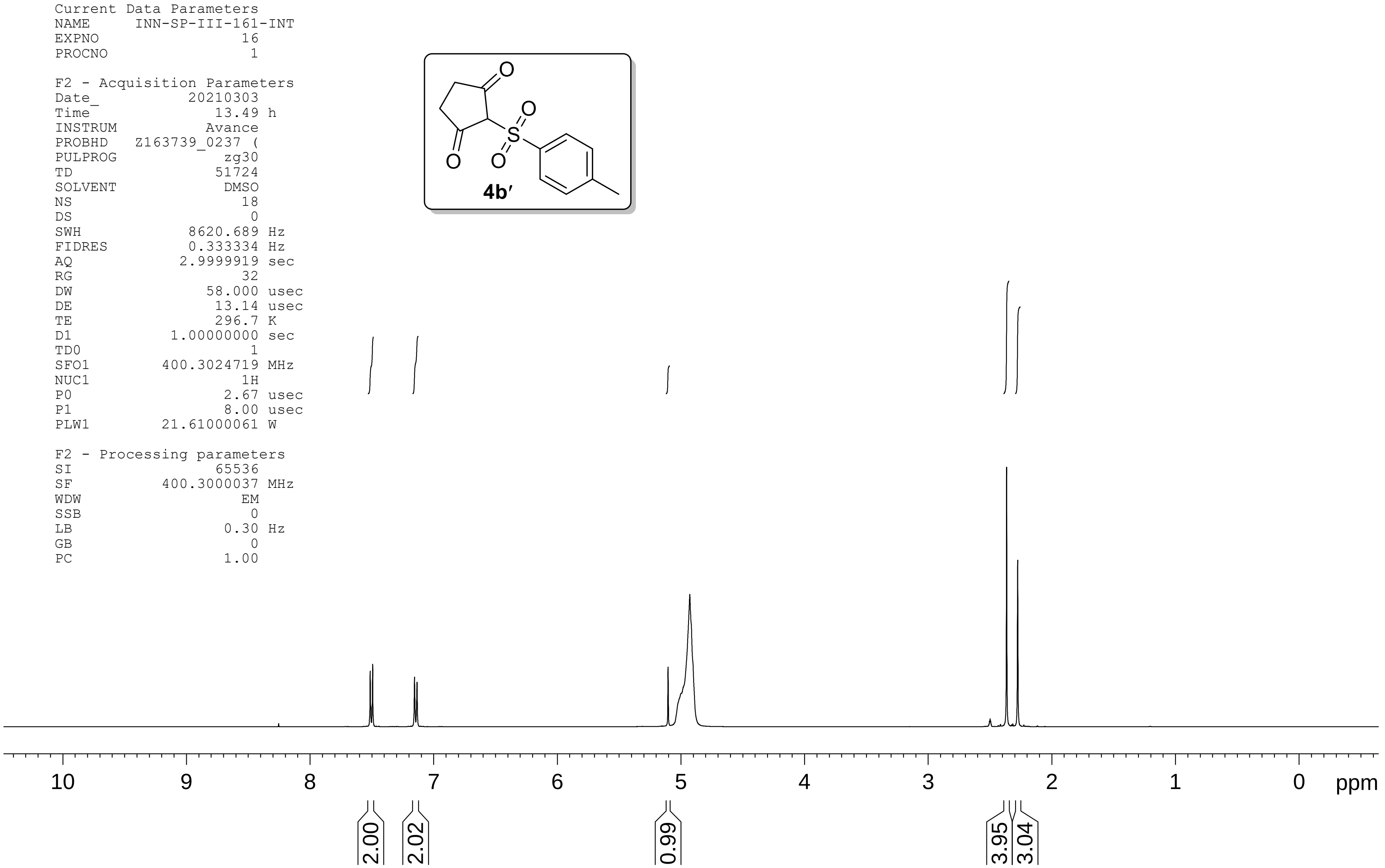

Figure S34: ^{1}H NMR spectrum of 4b'.

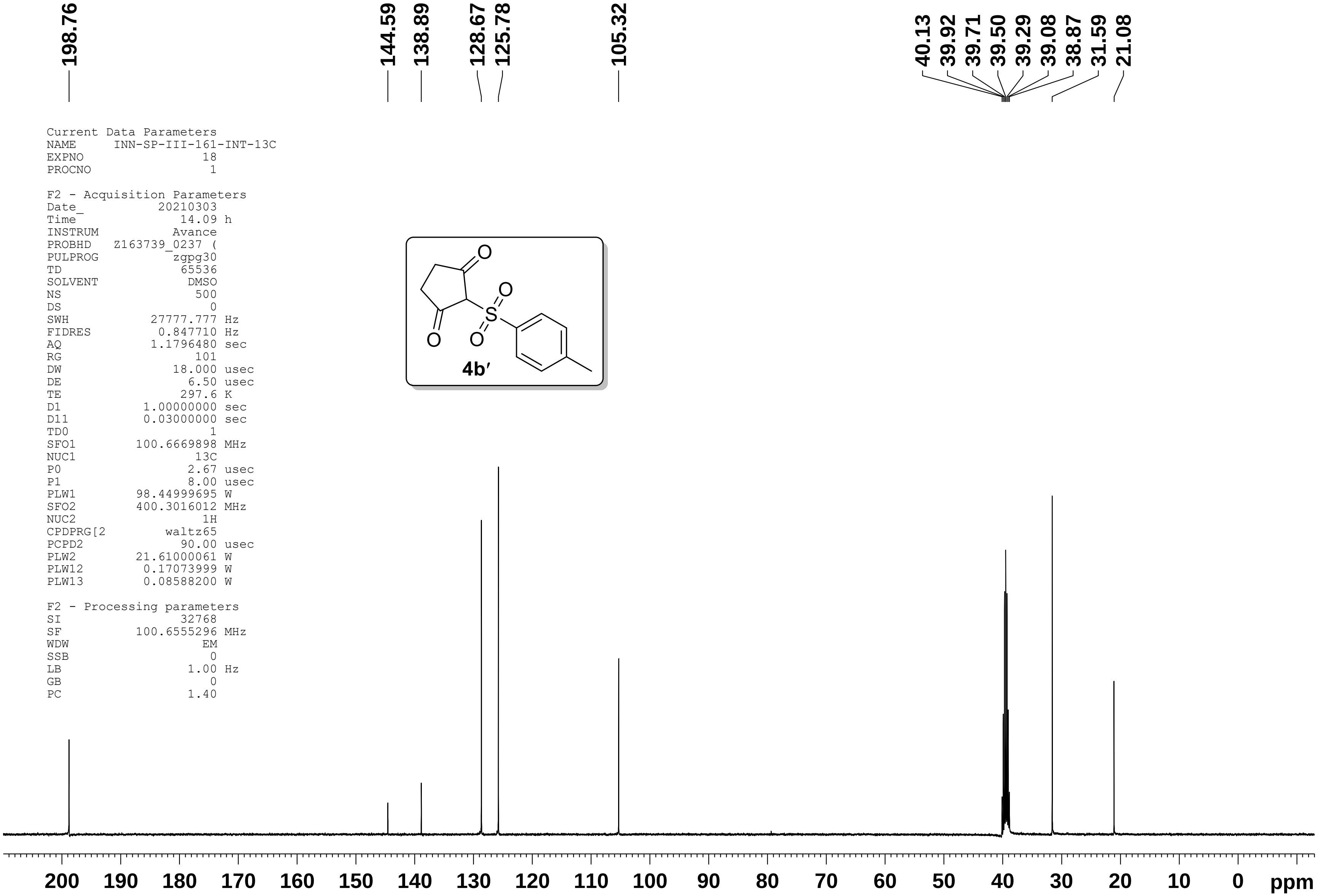

Figure S35: ^{13}C NMR spectrum of 4b'.

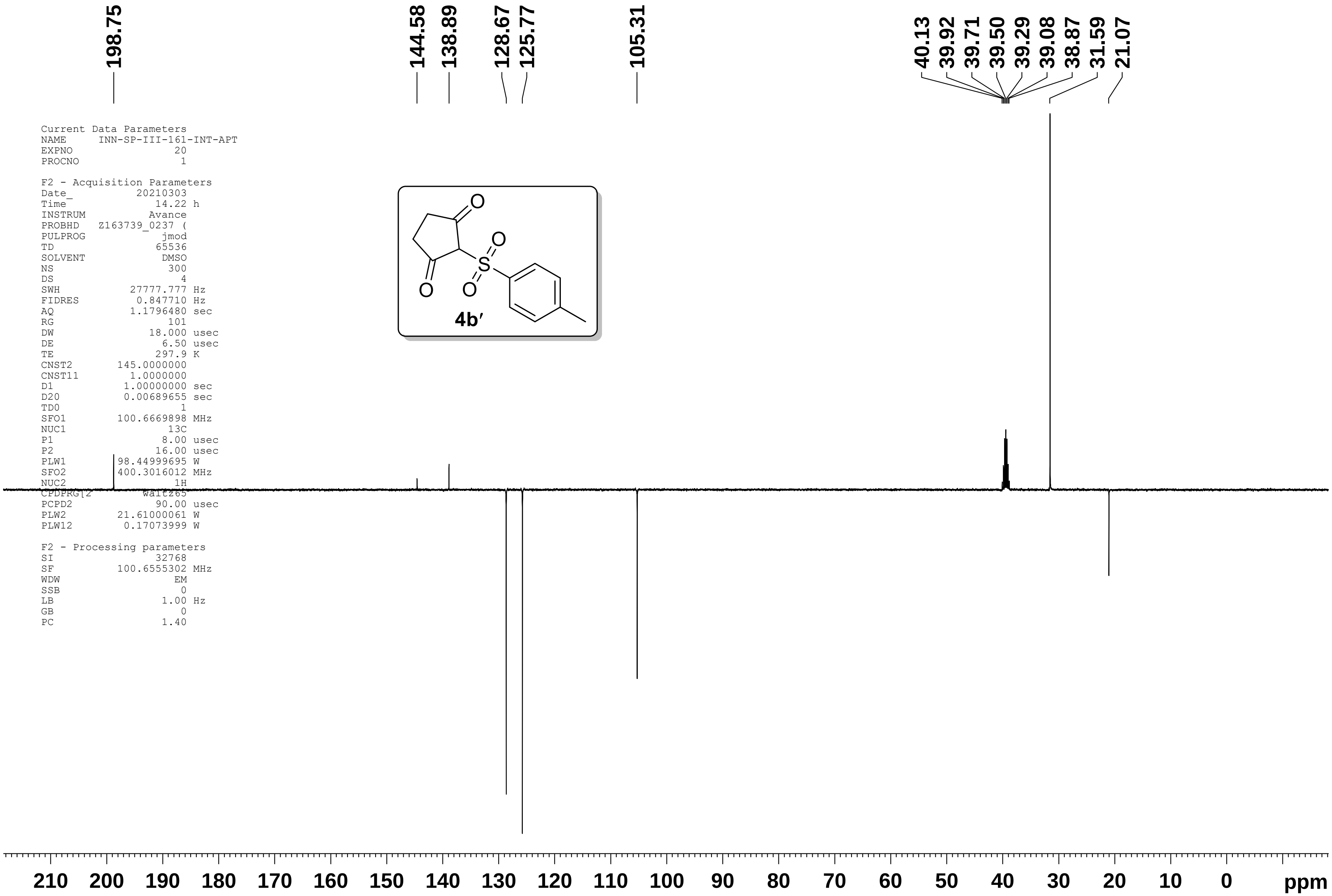

Figure S36: ^{13}C-APT NMR spectrum of 4b'.

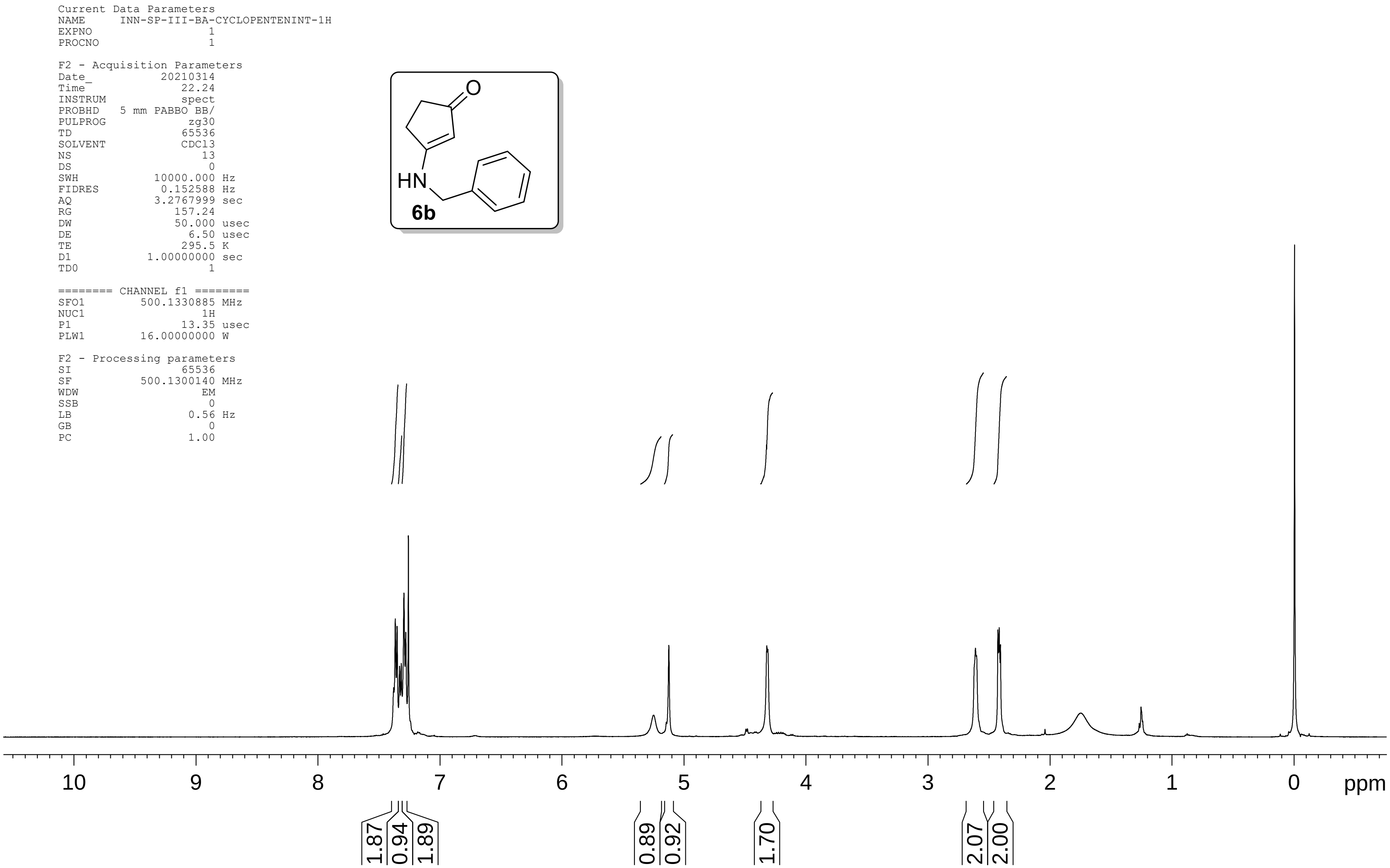

Figure S37: ^{1}H NMR spectrum of 6b.

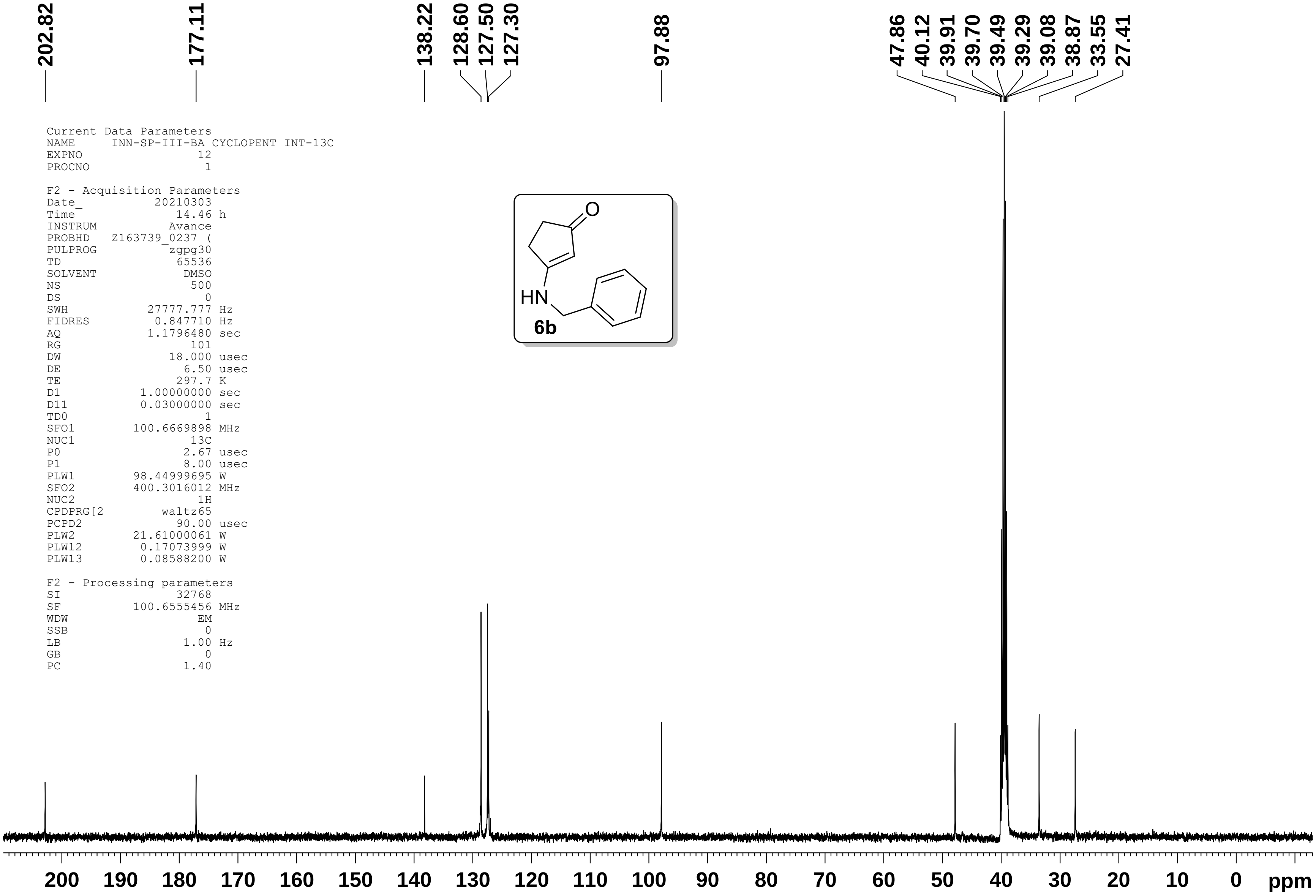

Figure S38: ^{13}C NMR spectrum of 6b.

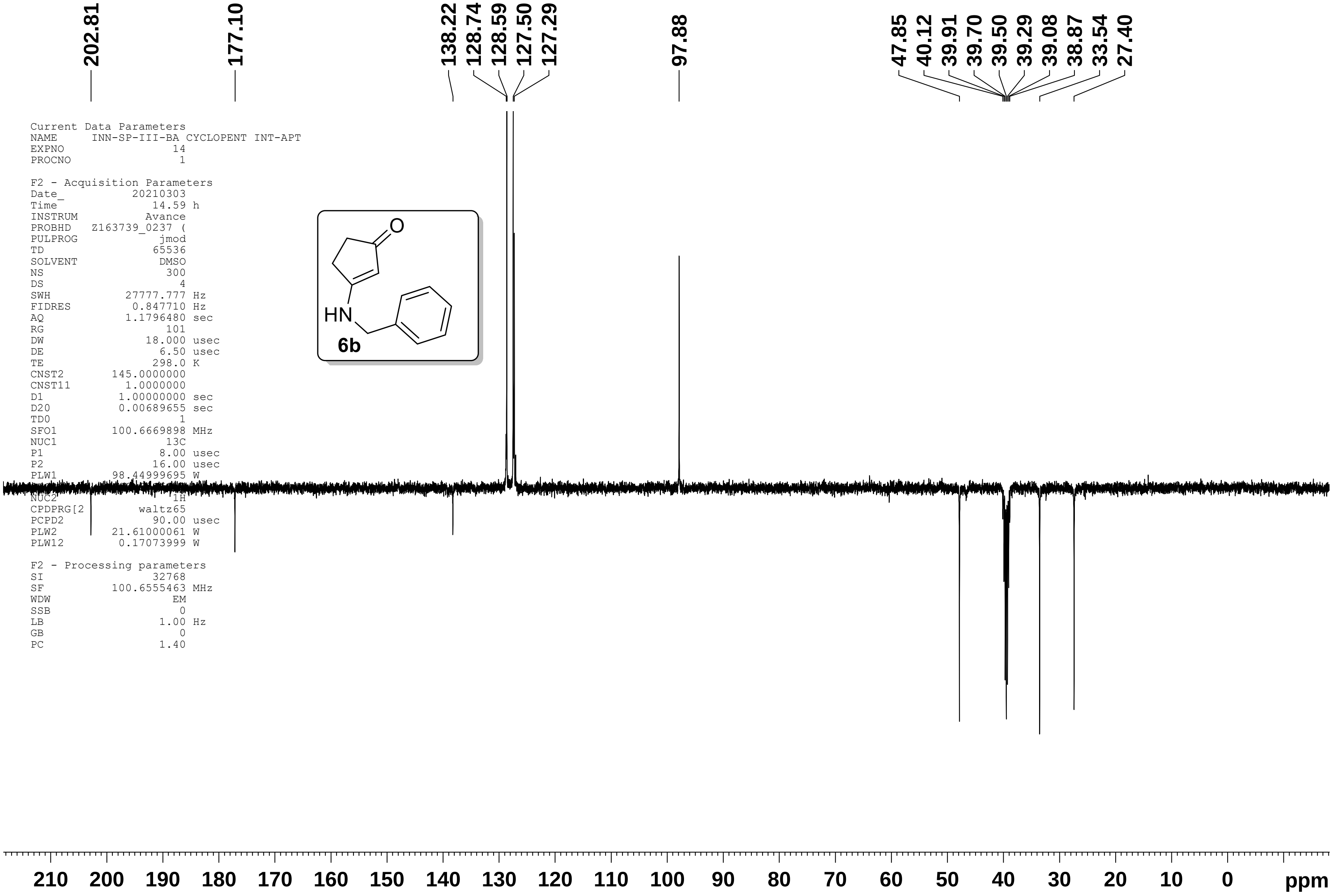

Figure S39: ^{13}C-APT NMR spectrum of 6b.

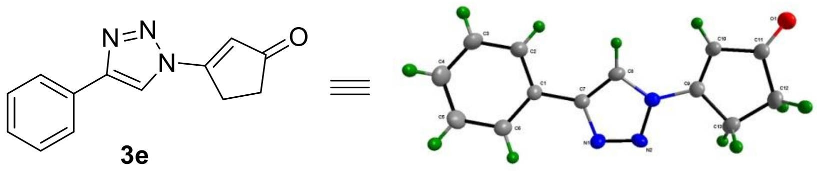

3e

Figure S40: ORTEP diagram of 3e.

Table S1: Crystal table of 3e.

Identification code	Reflection List
Empirical formula	$C_{13}H_{11}N_3O$
Formula weight	225:25
Temperature/K	293(2)
Crystal system	monoclinic
Space group	$P2_1/n$
a/Å	5.890
b/Å	24.263
c/Å	7.661
α/°	90
β/°	99.67
γ/°	90
Volume/Å^3	1079.3
Z	4
ρ_{calc}g/cm^3	1.386
μ/mm^{-1}	0.092
F(000)	472.0
Crystal size/mm^3	0.230 × 0.110 × 0.050
Radiation	MoKα (λ = 0.71073)
2□ range for data collection/°	6.354 to 49.992

Index ranges	? ≤ η ≤ ?, ? ≤ κ ≤ ?, ? ≤ λ ≤ ?
Reflections collected	1881
Independent reflections	1881 [R_{int} = 0.0876, R_{sigma} = 0.1717]
Data/restraints/parameters	1881/0/154
Goodness-of-fit on F^2	0.960
Final R indexes [I>=2σ (I)]	R_1 = 0.0511, wR_2 = 0.1082
Final R indexes [all data]	R_1 = 0.1056, wR_2 = 0.1493
Largest diff. peak/hole / e $Å^{-3}$	0.58/-0.69
